# Assessing the Efficacy of Monovalent and Commercialized Antivenoms for Neutralizing Moroccan Cobra *Naja haje* Venom: A Comparative Study

**DOI:** 10.3390/tropicalmed8060304

**Published:** 2023-06-02

**Authors:** Soukaina Khourcha, Ines Hilal, Iatimad Elbejjaj, Mehdi Karkouri, Amal Safi, Abdelaziz Hmyene, Naoual Oukkache

**Affiliations:** 1 Laboratory of Venoms and Toxins, Pasteur Institute of Morocco, Casablanca 20360, Morocco; drineshilal@gmail.com (I.H.); naoual.oukkache@pasteur.ma (N.O.); 2 Laboratory of Biochemistry, Environment and Food Technology, Faculty of Sciences and Technologies of Mohammedia, Hassan II University, Mohammedia 20650, Morocco; amalsafi88@gmail.com (A.S.); hmyeneaziz2002@yahoo.fr (A.H.); 3 Laboratory of Pathological Anatomy, University Hospital Center Ibn Rochd, Casablanca 20250, Morocco; madowine.elbejaj@gmail.com (I.E.); m.karkouri@chucasa.ma (M.K.)

**Keywords:** *Naja haje*, cobra venom, toxicity, physiopathology, histology, antivenom neutralization

## Abstract

In Morocco, eight species of venomous snakes belonging to the *Viperidae* and *Elapidae* families are responsible for severe envenomation cases. The species from the *Elapidae* family is only represented by the medically relevant cobra *Naja haje*, which is widely distributed in North Africa. However, there is little information on the systemic effects of Moroccan cobra venom on vital organs due to regional variations. It has been demonstrated that the venom of *Naja haje* from Egypt causes hemorrhage, while the venom of the Moroccan cobra is neurotoxic and devoid of systemic bleeding. This variability is known to significantly influence treatment efficacy against *Naja haje* cobra bites in the Middle East. In this study, we examined the pathophysiological mechanisms responsible for the lethality induced by *Naja haje* venom, as well as the evaluation of the neutralizing capacity of two antivenoms; the monospecific antivenom made for *Naja haje* only and the antivenom marketed in the Middle East and North Africa. We first determined the toxicity of *Naja haje* venom by LD_50_ test, then compared the neutralizing capacity of the two antivenoms studied by determining the ED_50_. We also performed histological analysis on Swiss mice envenomed and treated with these antivenoms to observe signs of cobra venom envenomation and the degree of reduction of induced systemic alterations. The results showed significant differences between both antivenoms in terms of neutralization. The monospecific antivenom was four times more effective than the marketed antivenom. These results were confirmed by a histological study, which showed that monospecific antivenoms neutralized severe signs of mortality, such as congestion of blood vessels in the heart and kidneys, pulmonary and renal edema, cytoplasmic vacuolization of hepatocytes in the liver, and infiltration of inflammatory cells in the brain and spleen. However, the polyvalent antivenom failed to protect all severe lesions induced by *Naja haje* venom in mice. These findings highlight the negative impact of geographic variation on the effectiveness of conventional antivenom therapy and confirm the need for a specific *Naja haje* antivenom for the effective treatment of cobra envenomation in Morocco.

## 1. Introduction

Snakebite envenomation is a significant public health issue, especially in tropical and developing countries, where its management is often inadequate. The World Health Organization (WHO) classified snakebites as a “priority neglected tropical disease” in 2017 due to their severity [1]. Most snakebites (95%) occur in rural areas, leading to up to 125,000 deaths annually and three times as many amputations and permanent disabilities [1,2,3]. In Africa alone, over 1 million snakebites are recorded annually, resulting in over 100,000 envenomations and over 10,000 fatalities [4,5,6]. Among the venomous snakes in the region, cobras (*Naja* sp.) are common and capable of delivering deadly venom. Cobras, such as the Egyptian cobra “*Naja haje*” and the spitting cobra “*Naja nigricollis*” are among the most dangerous and widespread species in North Africa and Africa, respectively. Their venom is a complex mixture of proteins and enzymes with varying toxicity and antigenicity, influenced by geographical location and environmental conditions. These variations in venom composition highlight the need for region-specific antivenoms to combat snakebite envenomations effectively [7,8].

In the North African region, the cobra species *Naja haje* poses a significant threat due to its variable toxicity and wide distribution. It is found across multiple countries, such as Algeria, Egypt, Libya, and Morocco, and is responsible for causing substantial morbidity and mortality through its venom’s potent three-finger toxins (3FTxs). These neurotoxins can rapidly induce neuromuscular paralysis and respiratory failure by binding to nicotinic acetylcholine receptors in skeletal muscles. Additionally, the venom contains phospholipases A_2_ (PLA_2_), which can lead to tissue necrosis and permanent disabilities [9,10,11]. Other components of cobra venom, such as hyaluronidases (HYA), metallo- (SVMP) and serine proteinases (SVSP), secretory proteins rich in cysteine (CRiSPs), L-amino acid oxidases (LAAOs), phosphodiesterases (PDEs), acetylcholinesterases (AChEs), nucleases (NUCs), hyaluronidases (HYA), nerve growth factors (NGFs), and venom factors cobra (CVFs), may have hemorrhagic, anticoagulant [12], pro-inflammatory [13,14], or immunogenic [15] properties [16]. Envenomation by *Naja haje* can result in systemic neurotoxicity and local tissue necrosis, leading to severe symptoms such as abdominal pain, vomiting, convulsions, blood clotting disorders, and cardiorespiratory impairment. Prompt administration of specific antivenom is crucial for neutralizing the venom toxins and preventing further harm to the victim [17,18,19].

Effective management of snakebites requires a comprehensive approach, including prevention, early detection, and timely access to appropriate treatment [20,21]. The approach must involve collaboration between healthcare providers, researchers, and policymakers to develop and implement effective strategies for managing this neglected tropical disease. These strategies may include developing and testing new antivenoms, improving diagnostic tools, and training healthcare providers on appropriate management protocols. By working together, we can reduce the number of snakebite-related deaths and disabilities and improve the quality of life for affected communities. To enhance the efficacy of current treatments and effectively reduce the morbidity and mortality associated with cobra envenomation in Morocco, our work aims to investigate the toxicity of Moroccan cobra *Naja haje* venom. Specifically, we will examine the resulting physiopathological alterations in mice envenomated with a sublethal dose of the venom and evaluate the capacity of two types of antivenoms to neutralize venom-induced alterations. One of the antivenoms is a monospecific antivenom (*NhMo_AV*) developed by our laboratory, while the other is a commercially available polyvalent antivenom (*Inoserp-MENA)* that is currently the only option for snakebite treatment in Morocco and was developed by the Inosan Institute in Mexico.

Furthermore, our study aims to investigate the potential benefits of developing specific antivenoms. This will provide us with valuable data on the toxicity and physiopathological profile of the venom and highlight the significance of developing specific antivenoms to face the major problem of venom variability. The insights obtained from this study will shed light on the mode of action of Moroccan Cobra venom. They will inform the potential development of specific antivenoms against the venom implicated in severe cases of envenomation in Morocco. Ultimately, our research has the potential to significantly improve patient outcomes and reduce the burden of cobra envenomation in Morocco. By providing a better understanding of the toxicity and physiopathology of the venom and evaluating the efficacy of different antivenoms, we hope to develop more effective and targeted treatments for cobra envenomation.

## 2. Materials & Methods

### 2.1. Venoms

**Figure 1:**
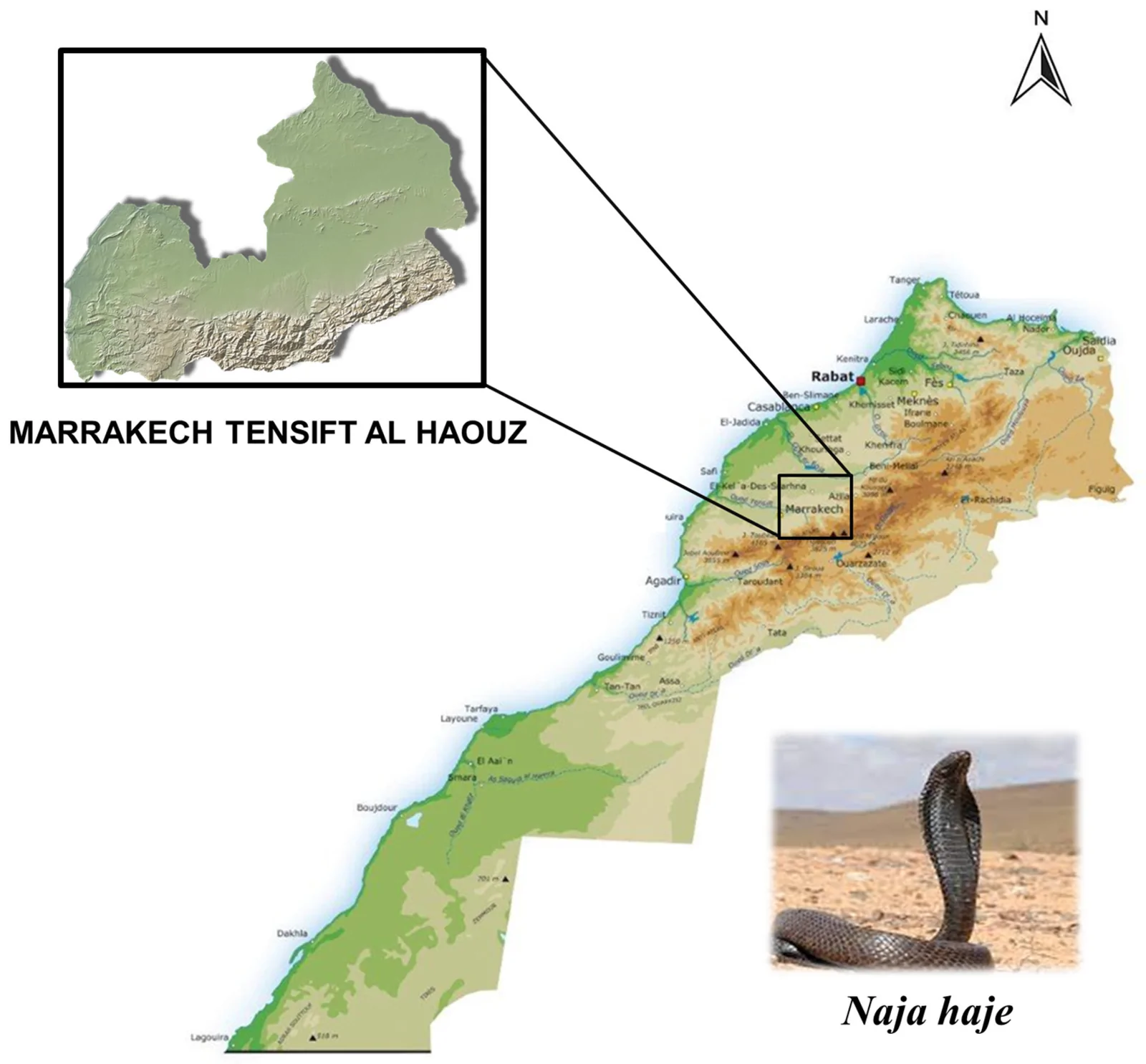
Region where the Cobra specimens were collected.

The *Naja haje* venom was pooled from seven adult specimens collected from the surroundings of Marrakech, where severe envenomation cases were recorded following *Naja haje* bites (Figure 1) and then kept at the serpentarium of the Animal Unit of the Pasteur Institute of Morocco (PIM). Venoms were extracted from fangs after manual stimulation of the venom gland. The extracted venoms were recovered using distilled water and centrifuged at 13.000× *g* at 4 °C for 15 min for 10 min. The supernatants were lyophilized and then frozen at −20 °C until use.

### 2.2. Antivenoms

Commercial polyvalent antivenom *Inoserp-MENA* is prepared by immunizing horses with a mixture of venoms and provided by the Anti-Poison Control Center of Morocco (CAPM). The batch number of the *Inoserp-MENA* antivenom used in this study is 0IT09001, manufactured in September 2020, with an expiration date in September 2023. A monospecific antivenom against *Naja haje* venom *NhMo_AV* was generated at the Pasteur Institute of Morocco using rabbits. The antivenom production involved subcutaneous inoculation of rabbits at three points near the neck, close to the lymphatic system. Lyophilized serpent venom powder aliquots were dissolved in physiological saline, and the immunogens were prepared by mixing diluted venom solutions with complete Freund’s adjuvant for the initial immunization, and incomplete Freund’s adjuvant for booster immunizations. To ensure production control, blood samples were collected from marginal ear veins and allowed to coagulate at room temperature. The resulting serum was then separated through centrifugation and used for monitoring antibody formation [22,23]. Immunoglobulins were isolated from the serum using the main protein purification steps [24]. F(ab′)_2_ fragment concentrations were determined spectrophotometrically using an extinction coefficient (ε) of 1.4 for a 1 mg/mL protein concentration at 280 nm using a 1 cm light pathlength cuvette.

### 2.3. SDS-Page and Densitometric Analysis

SDS-polyacrylamide gel electrophoresis (SDS-PAGE) was conducted according to the method of Laemmli [25], using broad range protein marker (14.4 to 97 kDa) as molecular weight calibration. Venom sample *Naja haje* (20 µg) was loaded into a 12% gel and the electrophoresis was performed under reducing conditions at 90 V for 2.5 h. Gel was stained with Coomassie Brilliant Blue R-250 and destained on methanol/acetic acid solution in water (30/10, *v*/*v*). The relative abundance of each band was determined by densitometric analysis (% band intensity) using the Gel Analyzer program.

### 2.4. Toxic Activities of Naja haje Venom

#### 2.4.1. Lethality

The lethal activity of *Naja haje* venom is evaluated according to the determination of the Median Lethal Dose (LD_50_). The intraperitoneal injection was carried out at the animal clinic of the Pasteur Institute of Morocco. Six groups of four mice 18–22 g (Swiss strain male) were injected with increasing quantities of 0.1–3 µg/g for the intraperitoneal ‘IP’ and intravenous ‘IV’ route. A control group of four mice was injected with 500 μL of physiological solution. The mortality was recorded after 24–48 h and the results were calculated using the GraphPad Prism 7.0 software through non-linear regression (variable slope) [26].

#### 2.4.2. Experimental Envenomation

An experimental envenomation by the *Naja haje* venom was carried out on two groups (*n* = 4, 20 ± 2 g). The mice were injected with a sublethal dose of the venom *Naja haje* (1/2 LD_50_), while the control groups received 500 µL of physiological saline (0.9%). The mice were then sacrificed after 6 h after administration of venom and the brain, heart, lungs, kidneys, and liver were removed and placed in a solution of Formol 10%.

### 2.5. Neutralization of Toxic Activities

#### 2.5.1. Median Effective Dose (ED_50_) Determination

The protocol for neutralizing venom by antivenoms has been used following WHO recommendations [21]. A challenge dose of the venom 5LD_50_ and 3LD_50_ was mixed, respectively with various dilutions by the monovalent Moroccan antivenom (*NhMo_AV*) and commercialized polyvalent antivenoms (*Inoserp-MENA*) to give a total volume of 500 μL. The mixture was incubated at 37 °C for 30 min and then injected intraperitoneally into the mice (18–22 g, *n* = 6 per dose). The mice were allowed free access to food and water, the survival ratio was recorded at 24 h and 48 h post-injection. The neutralization capacity of both antivenoms, *NhMo_AV* and *Inoserp-MENA*, was assessed using several parameters. The ED_50_ (Effective Dose 50) was calculated as the amount of reconstituted antivenom that resulted in 50% survival in animals challenged with venom. The ER_50_ (Effective Ratio 50) was determined as the amount of venom (in mg) neutralized per ml of antivenom at which 50% of envenomed mice survived. Additionally, the neutralization potency (P) was defined as the amount of venom completely neutralized by a unit volume of antivenom (mg/mL). The measurements ED_50_ were processed using non-linear regression analysis, specifically utilizing a variable slope dose–response curve, with the assistance of GraphPad Prism 7.0 software.

#### 2.5.2. Neutralization of the Alterations Induced by *Naja haje* Venom

Three experiments were conducted to study the efficacy of both the antivenoms monovalent Moroccan *Naja haje* (*NhMo_AV*) and commercialized polyvalent (*Inoserp-MENA)* to neutralize the physiopathological effect induced by cobra venom in the vital organs. Each experiment consisted of three groups of mice (four per group). In Group I, the mice were administered with the complexation method, which involved pre-incubating a sublethal dose of *Naja haje* venom with antivenom and a median effective dose of monovalent antivenom (*NhMo_AV*). Group II and III, the mice received, respectively both antivenoms (ED_50_) after 2 h of venom injection. The mice of the negative control group were injected with an ‘IP’ route of physiological solution. All the groups of mice were sacrificed 6 h after administration of antivenom and the organs brain, heart, lungs, liver, kidneys, and spleen were removed for histological study.

### 2.6. Histological Study

The vital organs (brain, heart, lungs, liver, kidneys, and spleen) were cautiously harvested for histological evaluation and fixed immediately in a 10% formaldehyde solution. After 24 h of fixation, the tissues were dehydrated in ethanol, cleared in xylene, impregnated, and embedded in paraffin wax. Then, transverse sections of 4 µm in thickness were prepared and stained with Hematoxylin and Eosin for histopathological examinations [27,28].

### 2.7. Animals and Ethics Statement

The toxic activity of *Naja haje* venom and the neutralizing effect of a monospecific antivenom anti-*Naja haje* and commercial antivenom were performed in 18–22 g male Swiss C57/BL6 mice, which were obtained from the central animal house, provided by the animal unit of the Pasteur Institute of Morocco. The protocol of the animal studies was based on the ethical guidelines adopted by the WHO [29] and approved by a local ethics committee of the Institut Pasteur of Morocco, under agreement number 8.3.A-2015.

1. Standard cages (floor area):

Mice were housed in standard cages measuring 42.5 cm × 26.6 cm × 18.5 cm, corresponding to a total floor area of approximately 1130 cm^2^, with four animals per cage (approximately 282 cm^2^ per animal).

2. Humane endpoint and euthanasia method:

Animals were closely monitored throughout the experiments, and predefined humane endpoints were applied, including severe distress or moribund state.

Mice were euthanized by CO_2_ inhalation using a gradual-fill method, in accordance with current ethical recommendations. Death was confirmed by the absence of respiratory movements and lack of response to external stimuli.

### 2.8. Statistical Analysis

Values were calculated as mean ± standard error (SE) of the mean of three to five experiments. All statistical analyses and data presentations were generated using GraphPad Prism 7.0 software. For all statistical tests, *p* < 0.05 was considered statistically significant.

## 3. Results

### 3.1. SDS-Page Analysis

**Figure 2:**
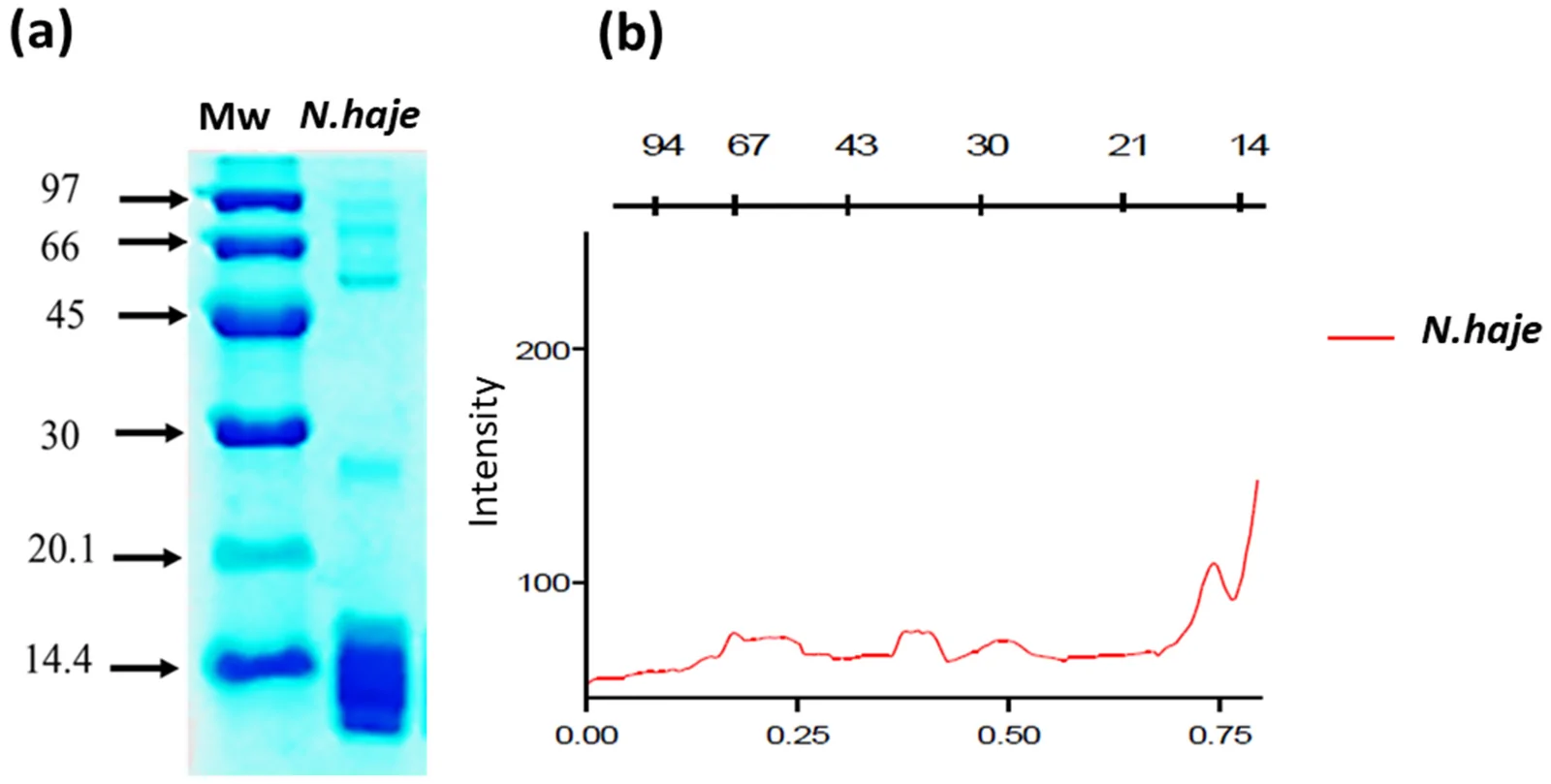
(**a**) The profile of SDS-PAGE electrophoresis at 12% of the crude venom of cobra *Naja haje* (20 μg). (**b**) Densitometric analysis of SDS-PAGE profiles venom under reducing conditions.

The protein profile analysis by SDS-PAGE of *Naja haje* venom by densitometry software revealed the presence of a high-intensity band with low molecular weight between 14 and 21 kDa which are mostly neurotoxic molecules. Two bands of low intensity the 30 to 43 kDa and the 43 to 67 kDa were also observed (Figure 2).

### 3.2. Toxic Activities of the Naja haje Venom

#### 3.2.1. Determination of the LD _50_

The lethal activity of cobra *Naja haje* venom was determined by the mortality rate recorded in mice by different routes. The LD_50_ values showed that the (IP) route 16.05 µg/mouse is almost just as efficient as the (IV) route 18.9 µg/mouse due to the presence of toxins (low molecular weight peptides), that make the *Naja haje* venom bioavailable in blood circulation regardless of the injection mode (Table 1).

#### 3.2.2. *Naja haje* Venom Induced Pathological Changes in Mouse Organs

**Figure 3:**
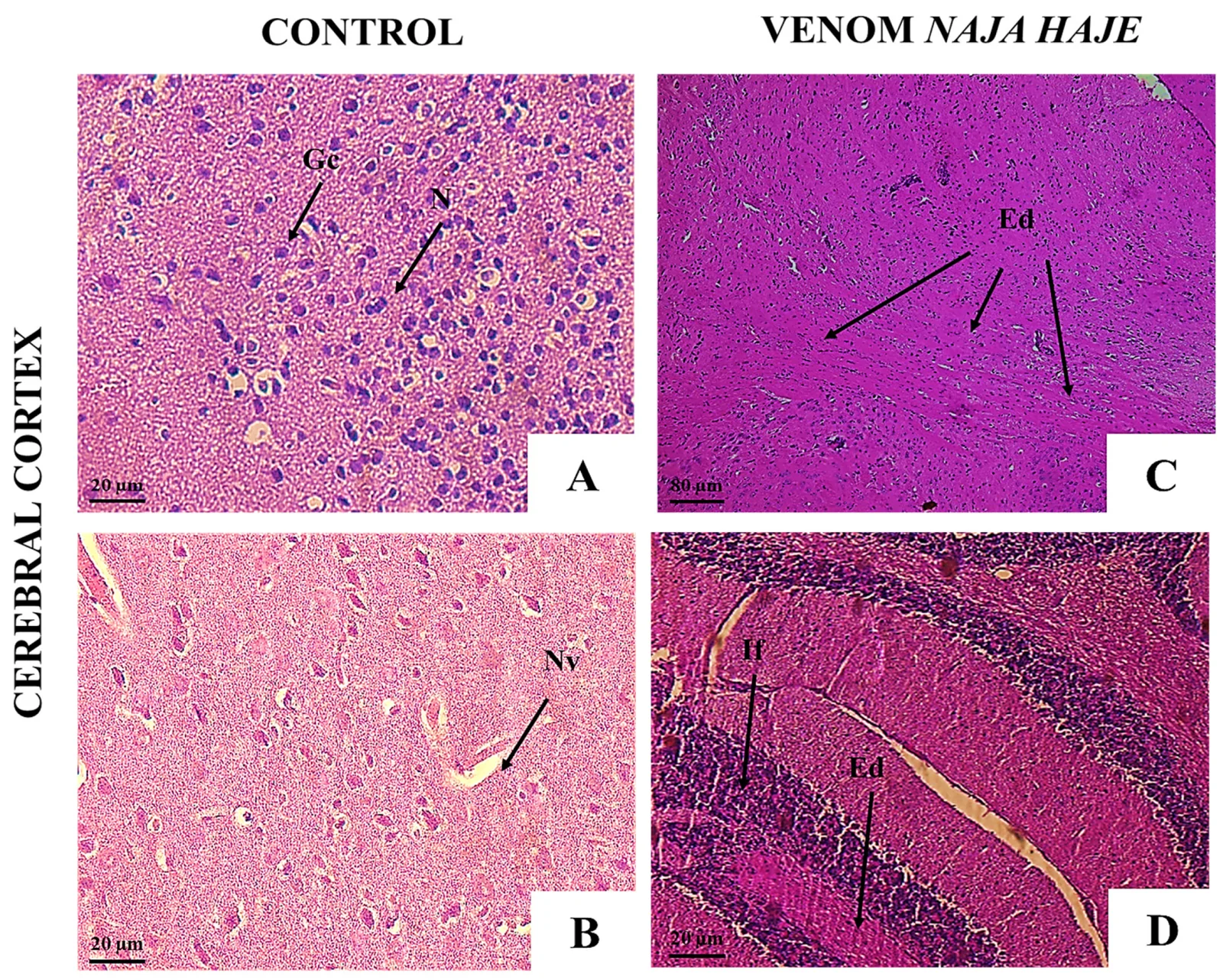
Histopathological examination caused by ½ LD_50_ of the *Naja haje* venom in mouse brain. (**A**,**B**): normal brain; **N**: Neurons appeared normal, **Gc**: Glial cells, **Nv**: Cerebral vessels. (**C**,**D**): effect of venom; neuronal degeneration; **Ed**: Edema, **If**: inflammatory infiltrate.

**Figure 4:**
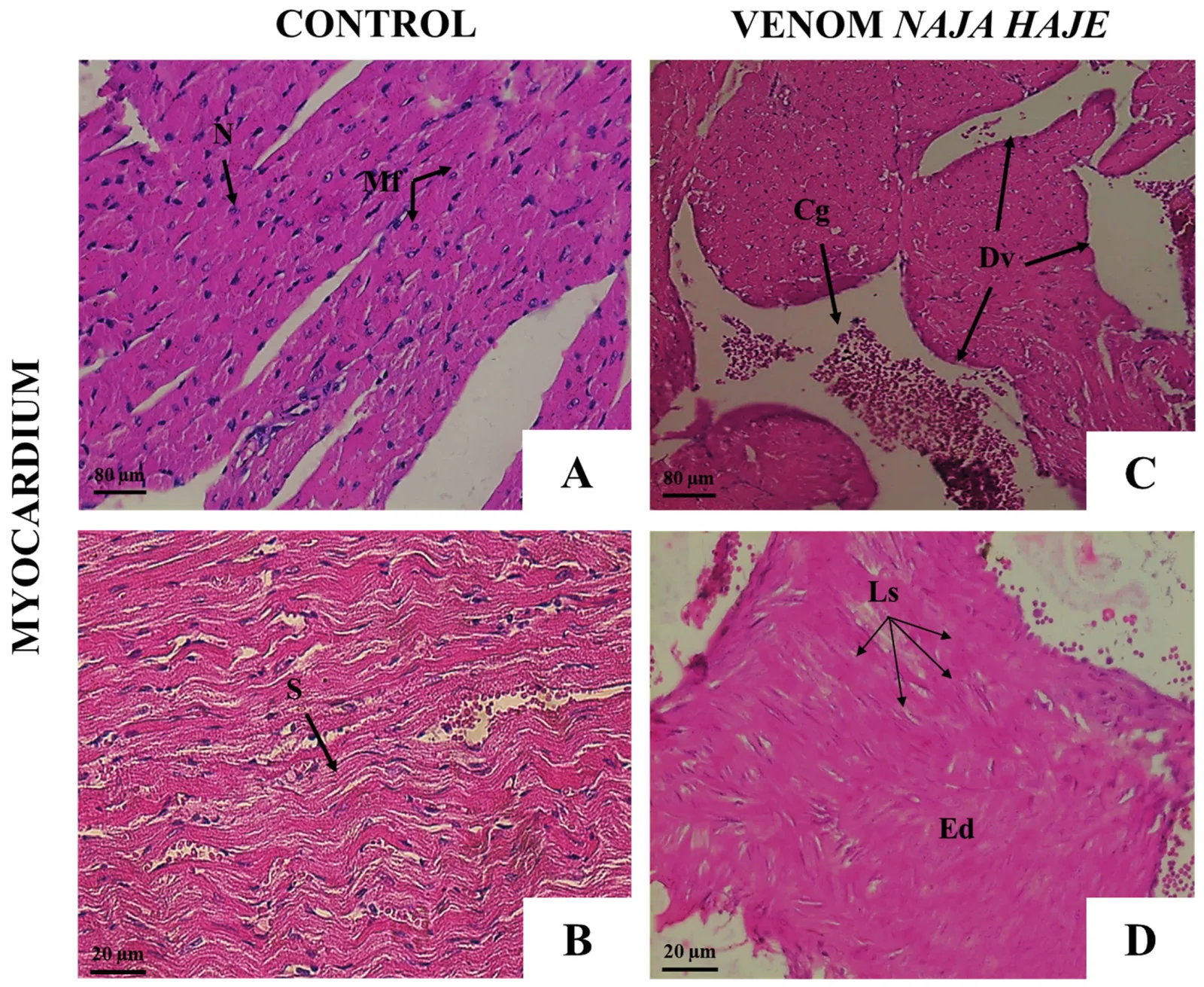
Histopathological changes in the heart tissue of envenomated mice by ½ LD_50_ of the *Naja haje* venom. (**A**,**B**): normal heart; **N**: nucleus appeared normal, **Mf**: muscle fibers, **S**: striations**,** (**C**,**D**): effect of venom *Naja haje*; degeneration of the cardiac muscles; **Cg**: congestion, **Dv**: vascular dilation, **Ed**: edema, **Ls**: loss of striations in the myocardial fibers.

**Figure 5:**
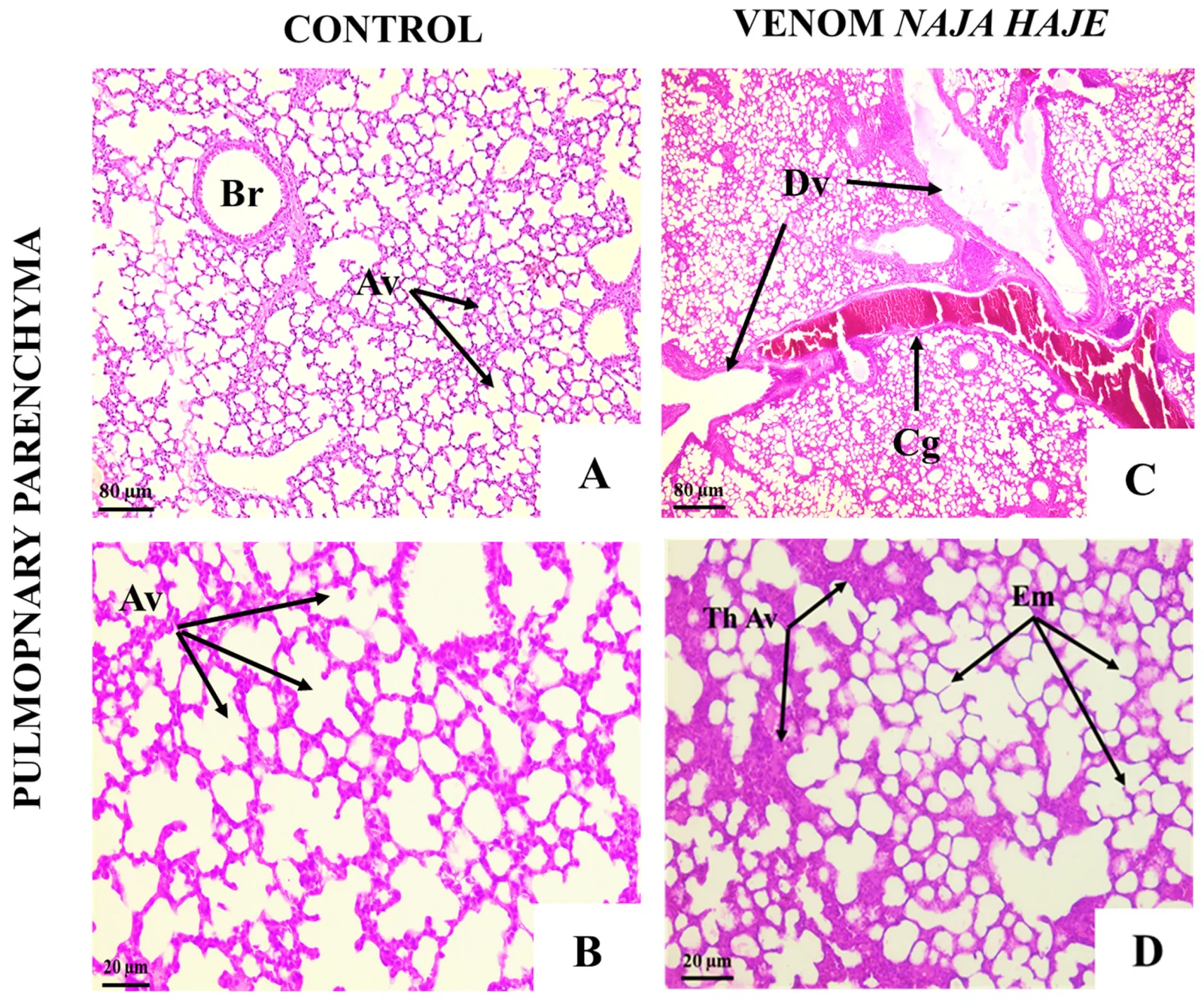
Histopathological changes in lung tissues of envenomated mice by ½ LD_50_ of the *Naja haje* venom. (**A**,**B**): Normal lung tissues; **Av**: Alveolus appeared normal, **Br**: Bronchioles. (**C**,**D**): Effect of venom *Naja haje*; degeneration of the pulmonary parenchyma; **Cg**: Congestion, **Dv**: Vascular dilation, **ThAv**: Thickening of the alveolar walls, Em: Emphysema.

**Figure 6:**
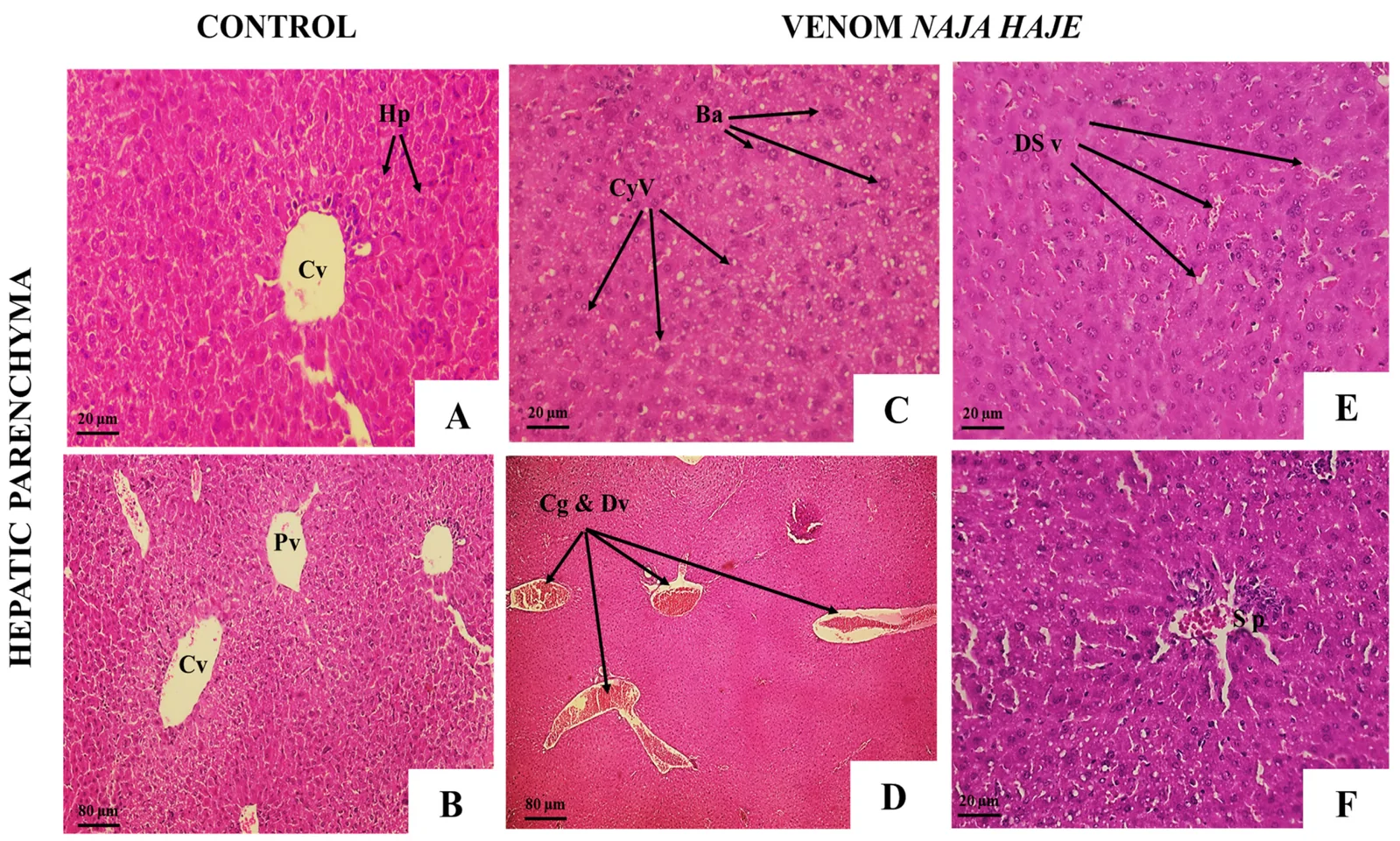
Histopathological changes in the liver tissues of envenomated mice by ½ LD50 of the Naja haje venom. (**A**,**B**): Normal liver tissues; **Hp**: hepatocyte appeared normal, **Cv**: Centrilobular vein, **Pv**: Portal vein. (**C**–**F**): Effect of venom Naja haje; degeneration of the hepatic parenchyma; **Cg**: Congestion, **Dv**: Vascular dilation, **Ba**: Ballooning of hepatocytes, **CyV**: Cytoplasmic vacuolation of hepatocyte, **DSv**: Dilation of the sinusoidal vessels.

**Figure 7:**
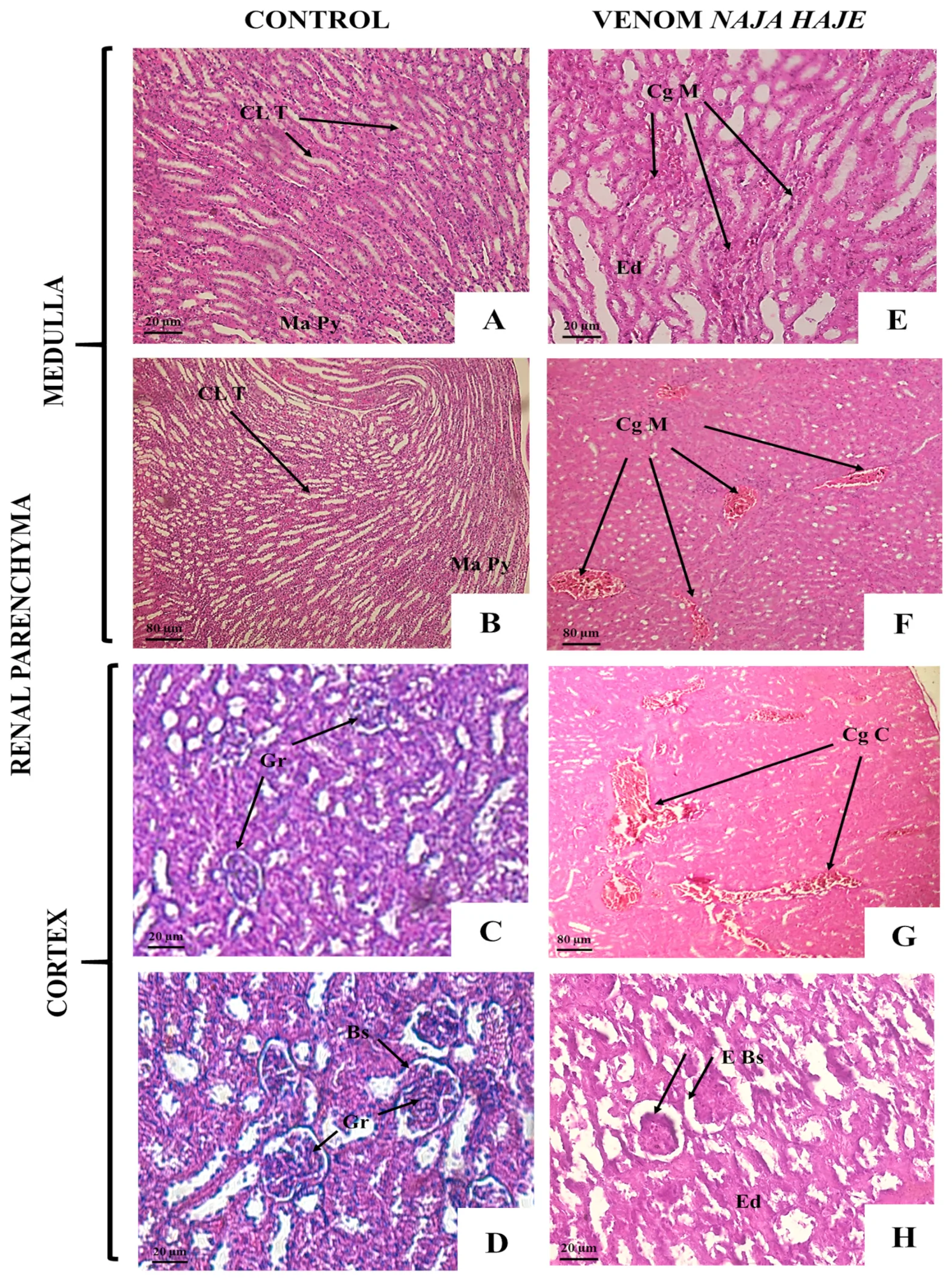
Histological changes in the kidney of envenomated mice by ½ LD_50_ of the *Naja haje* venom. (**A**,**B**): Normal renal medulla. (**C**,**D**): Normal renal cortex; **Gr**: Glomerulus, **Bs**: Bowman’s space, **CLT**: Collecting tubes. (**E**–**H**): Effect of venom *Naja haje*; degeneration of the renal parenchyma (cortex and medulla); **CgM**: Medullary congestion, **Ed**: Edema, **CgC**: Congestion of the cortical blood vessels, **EBs**: Enlargement of Bowman’s space.

**Figure 8:**
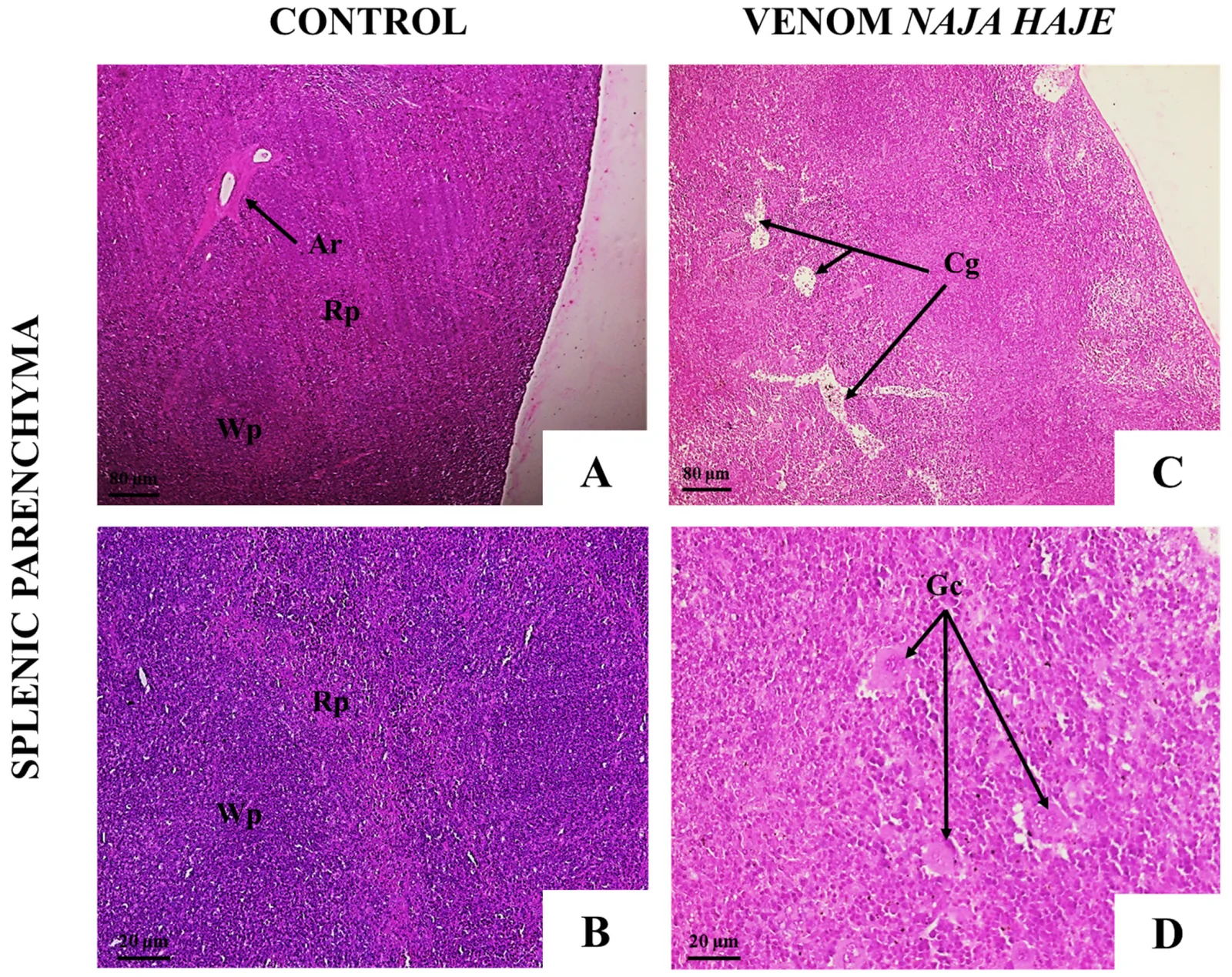
Histological changes in the spleen of envenomated mice by ½ LD_50_ of the *Naja haje* venom. (**A**,**B**): Normal splenic parenchyma, **Rp**: Red pulp, **Wp**: White pulp, **Ar**: Arteries. (**C**,**D**): Effect of venom *Naja haje*; degeneration of red and white pulp; **Cg**: Congestion, **Gc**: Giant cells.

The normal histological appearance was observed in all vital organs: kidneys, livers, hearts, lungs, and spleen after 6 h of injection of 500 µL of sterile 0.9% NaCl solution (Figure 3, Figure 4, Figure 5, Figure 6, Figure 7A–D and Figure 8A,B). Compared to control groups, mice envenomed with a sublethal dose (1/2 LD_50_) of *Naja haje* venom showed histopathological changes in the organs studied (Figure 3, Figure 4, Figure 5, Figure 6C–F, Figure 7E–H and Figure 8C,D).

**At the cerebral level**

The brain tissue of the untreated envenomed mice showed an edema accompanied by a moderate infiltrate of inflammatory cells mainly leucocytes and an important congestion of blood vessels (Figure 3C,D and Table 2).

**At the cardiac level**

Histological examination of cardiac tissue revealed cellular hypertrophy and loss of striation in the myocardial fibers with severe interstitial edema taking a large space between the fibers. Significant congestion and dilation of blood vessels were also observed (Figure 4C,D and Table 2).

**At the pulmonary level**

*Naja haje* venom resulted in thickened alveolar walls, moderate interstitial edema, and polymorphic inflammatory cell infiltration. There was also focal emphysema and severe congestion with blood vessel dilation. Dilatation of the bronchi was also observed (Figure 5C,D and Table 2).

**At the hepatic level**

Histological evaluation of the liver tissue showed hepatocyte damage such as ballooning and cytoplasmic vacuolation. Dilatation of the sinusoidal vessels was also observed with an increased number of kupffer cells. Congestion and dilatation of the central veins with agglutinated mass in their lumen were also noted. The portal spaces were the site of a medium inflammatory infiltrate made essentially of polymorphic cells (Figure 6C–F and Table 2).

**At the renal level**

Histological study of the renal tissue revealed severe damage in the cortex and the medulla. There was dilatation of Bowman’s space consisting of a large space between Bowman’s capsule and the glomeruli. Severe edema in the cortex and medulla was observed. Distal and proximal tubules were congested and dilated and made of hypertrophic cells (Figure 7E–H and Table 2).

**At the spleen level**

Histological examination revealed follicular hyperplasia and the presence of multinucleated giant cells. Additionally, there were signs of congestion and moderate dilation of blood vessels (Figure 8C,D and Table 2).

### 3.3. Neutralization of Toxic Potency

#### 3.3.1. Determination of the Median Effective Dose (DE_50_)

The lethal effect of the venom was neutralized by incubating the mixture with the fixed dose of venom with different doses of both monovalent and polyvalent antivenoms. The monovalent Moroccan antivenom (*NhMo_AV*) was found to be four times more effective than the commercialized one (*Inoserp-MENA*), with ED _50_ values of 67, 2, and 100 µL/mouse, respectively (Table 3). The net amount of venom neutralized per unit volume of antivenom, as indicated by the parameter “Potency” (P), was also consistently higher with the monospecific antivenom (1.392 mg/mL) than with the commercialized antivenom (0.32 mg/mL) (Table 3).

#### 3.3.2. Neutralization of the Pathological Alterations by Monovalent and Polyvalent Antivenoms

**At the cerebral level**

**Figure 9:**
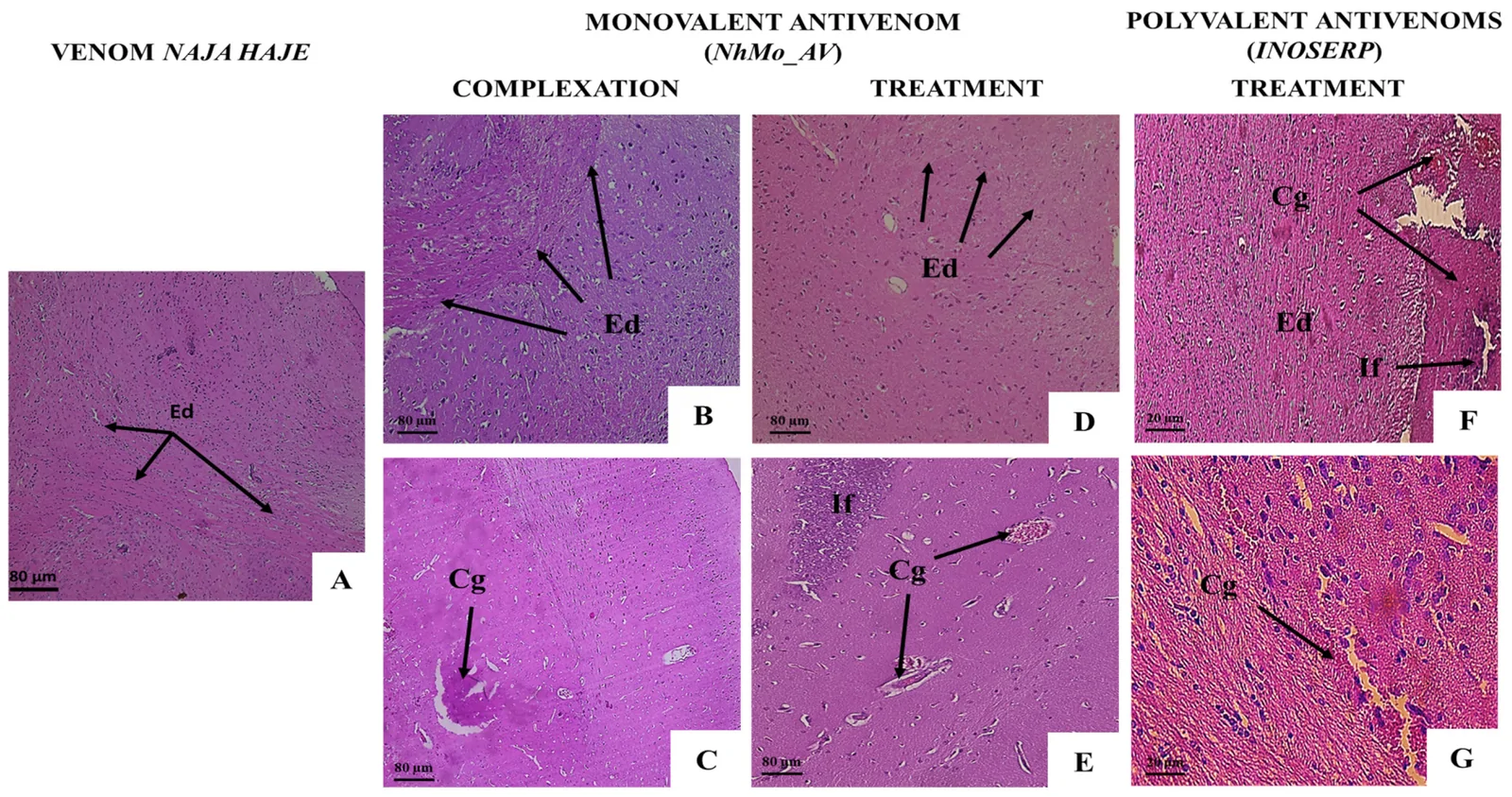
(**A**): Histopathological examination of brain tissue 6 h after intraperitoneal administration of *Naja haje* venom (½ LD_50_) in mice. (**B**,**C**): Protective effect of monovalent Moroccan antivenom (*NhMo_AV*) on venom-induced morphological changes by complexation method. (**D**,**E**): Envenomated mice treated with monovalent Moroccan antivenom (*NhMo_AV*) after 2 h of *Naja haje* venom injection. (**F**,**G**): Envenomated mice treated with commercialized polyvalent antivenom after 2 h of *Naja haje* venom injection. **Ed**: Edema, **Cg**: Congestion of blood vessels, **If**: inflammatory infiltrate.

The brains of mice in envenomed group I (Figure 9B,C and Table 2) and group II (Figure 9D,E and Table 2), treated two hours after injection of *Naja haje* venom, exhibited slight edema and focal congestion in certain blood vessels. Notably, group I showed a complete absence of inflammatory cells. However, histological sections from group III mice treated with commercial polyvalent antivenoms demonstrated persistent edema and vascular congestion. On the other hand, there was partial neutralization of the inflammatory infiltrate (Figure 9F,G and Table 2).

**At the cardiac level**

**Figure 10:**
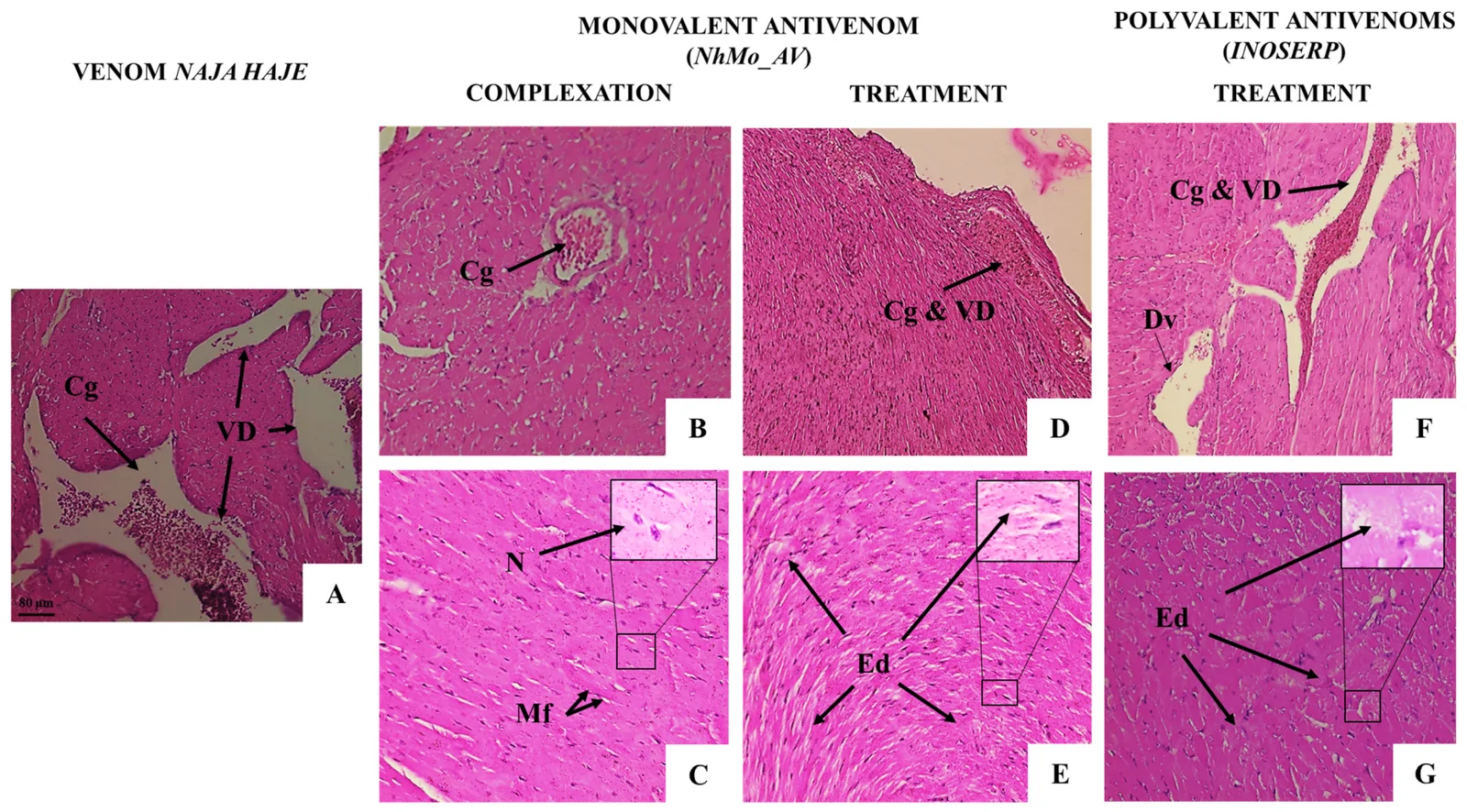
(**A**): Histopathological examination of cardiac muscle 6 h after intraperitoneal administration of *Naja haje* venom (½ LD_50_) in mice. (**B**,**C**): Protective effect of monovalent Moroccan antivenom (*NhMo_AV*) on venom-induced morphological changes by complexation method. (**D**,**E**): Envenomated mice treated with monovalent Moroccan antivenom (*NhMo_AV*) after 2 h of *Naja haje* venom injection. (**F**,**G**)**:** Envenomated mice treated with commercialized polyvalent antivenom after 2 h of *Naja haje* venom injection. **N**: Nucleus, **Mf**: Muscle fibers**, Cg**: Congestion, **VD**: vascular dilation, **Ed**: Edema.

Group I: Myocardial sections showed the absence of lesions such as interstitial edema, and a restoration of cardiac fiber striation. Additionally, blood vessel congestion and dilation were resolved and limited to specific focal areas (Figure 10B,C and Table 2).

Group II: Mice treated with monovalent antivenom (*NhMo_AV*) two hours after *Naja haje* venom injection exhibited mild edema with limited congestion in certain focal areas, while myocardial striation remained preserved (Figure 10D,E and Table 2).

Group III: Mice treated with the commercial polyvalent antivenom (*Inoserp-MENA*) two hours after *Naja haje* venom injection showed the presence of moderate interstitial edema with persistent congestion and dilation of blood vessels. However, the striation of myocardial fibers was conserved (Figure 10F,G and Table 2).

**At the pulmonary level**

**Figure 11:**
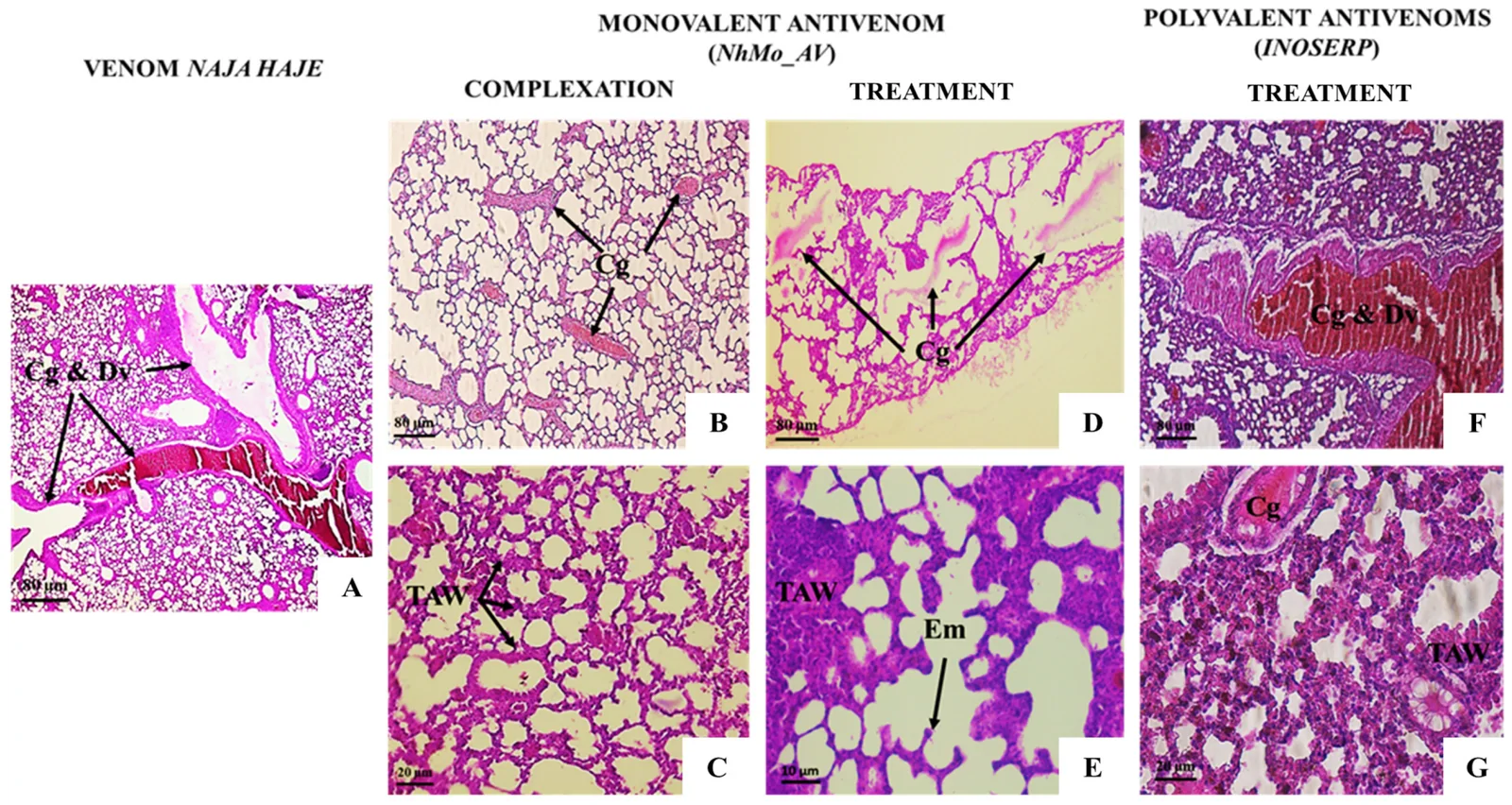
(**A**): Histopathological examination of lung tissue 6 h after intraperitoneal administration of *Naja haje* venom (½ LD_50_) in mice. (**B**,**C**): Protective effect of monovalent Moroccan antivenom (*NhMo_AV*) on venom-induced morphological changes by complexation method. (**D**,**E**): Envenomated mice treated with monovalent Moroccan antivenom (*NhMo_AV*) after 2 h of *Naja haje* venom injection. (**F**,**G**)**:** Envenomated mice treated with commercialized polyvalent antivenom after 2 h of *Naja haje* venom injection. **Cg**: Congestion, **Dv**: Vascular dilation, **ThAv**: Thickening of the alveolar walls, **Em**: Emphysema.

Group I: The lung tissue sections showed preserved lung tissue architecture with complete resorption of pulmonary edema. Mild thickening of the alveolar walls and congestion without dilation of blood vessels and bronchi were observed, accompanied by local emphysema (Figure 11B,C and Table 2).

Group II: Mice treated with monovalent antivenom (*NhMo_AV*) two hours after injection of *Naja haje* venom exhibited thickening of the alveolar walls and mild edema, along with a polymorphic inflammatory infiltrate. Partial neutralization of congestion and dilation of blood vessels and bronchi was observed (Figure 11D,E and Table 2).

Group III: Mice treated with commercial polyvalent antivenoms two hours after injection of *Naja haje* venom showed persistent signs of envenomation with partial neutralization of interstitial edema (Figure 11F,G and Table 2).

**At the hepatic level**

**Figure 12:**
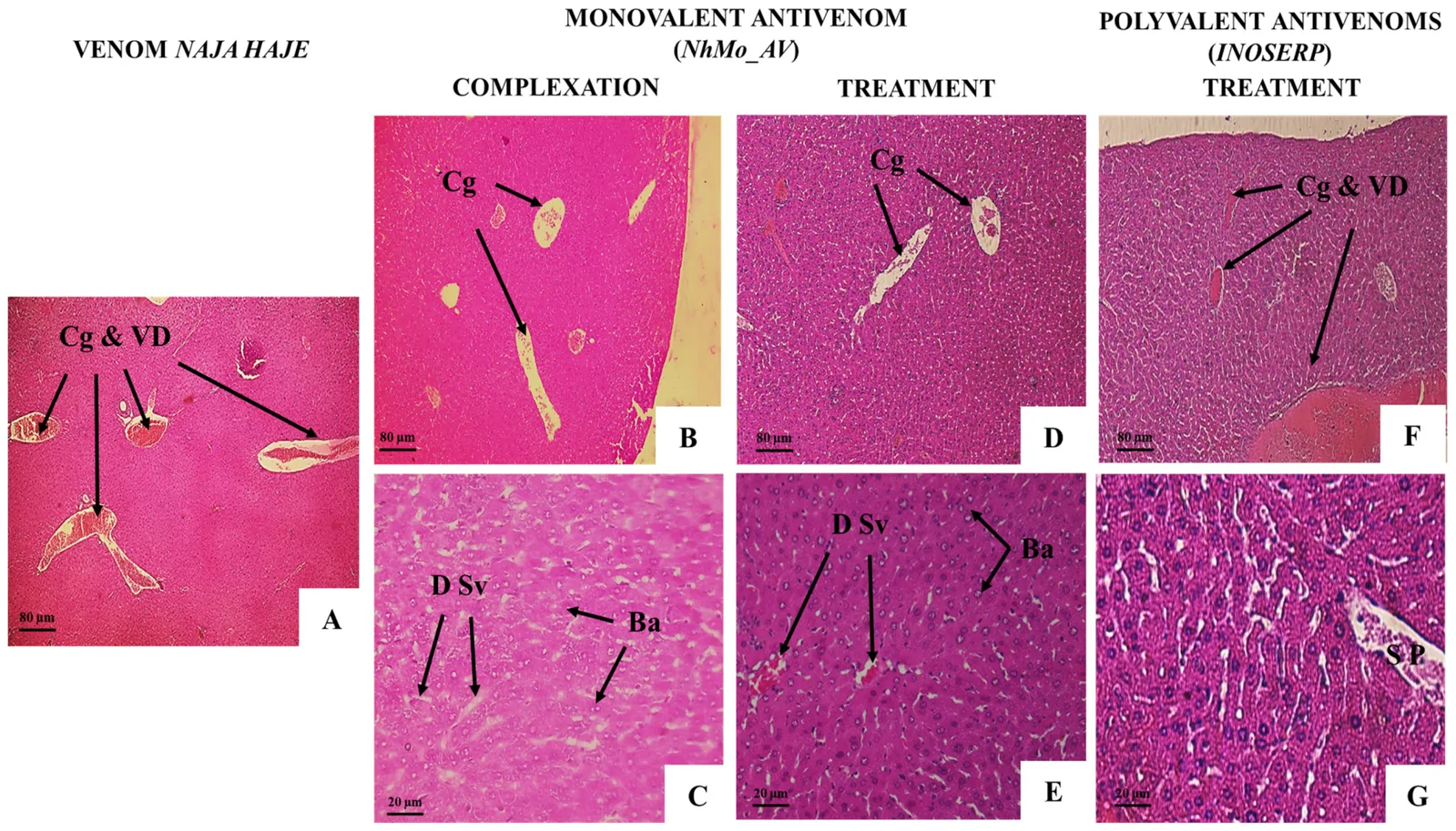
(**A**): Histopathological examination of hepatic tissue 6 h after intraperitoneal administration of *Naja haje* venom (½ LD_50_) in mice. (**B**,**C**): Protective effect of monovalent Moroccan antivenom (*NhMo_AV*) on venom-induced morphological changes by complexation method. (**D**,**E**): Envenomated mice treated with monovalent Moroccan antivenom (*NhMo_AV*) after 2 h of *Naja haje* venom injection. (**F**,**G**): Envenomated mice treated with commercialized polyvalent antivenom after 2 h of *Naja haje* venom injection. **Cg**: Congestion, **VD**: Vascular dilation, **Ba**: Ballooning of hepatocytes**, CyV**: Cytoplasmic vacuolation of hepatocytes, **DSv**: Dilation of the sinusoidal vessels.

Group I: Signs of improvement were observed in the liver tissue of this group. The liver architecture was preserved, with hepatocytes exhibiting focal ballooning without cytoplasmic vacuolation. Mild dilation of sinusoidal spaces and slight dilation of central and portal veins were also observed (Figure 12B,C and Table 2).

Group II: Histological sections of the liver showed similar lesions to those described in Group I. However, the cytoplasmic vacuolation of hepatocytes was moderately neutralized by monovalent antivenom (*NhMo_AV*). Additionally, there was a presence of polymorphic inflammatory cells (Figure 12D,E and Table 2).

Group III: Hepatic lesions persisted, with hepatocytes showing damage characterized by ballooning and cytoplasmic vacuolation. Dilation of porto-sinusoidal vessels and the presence of inflammatory infiltrates were observed. On the other hand, the congestion and dilation of central and portal veins were partially neutralized by the commercial polyvalent antivenom (*Inoserp-MENA)* (Figure 12F,G and Table 2).

**At the renal level**

**Figure 13:**
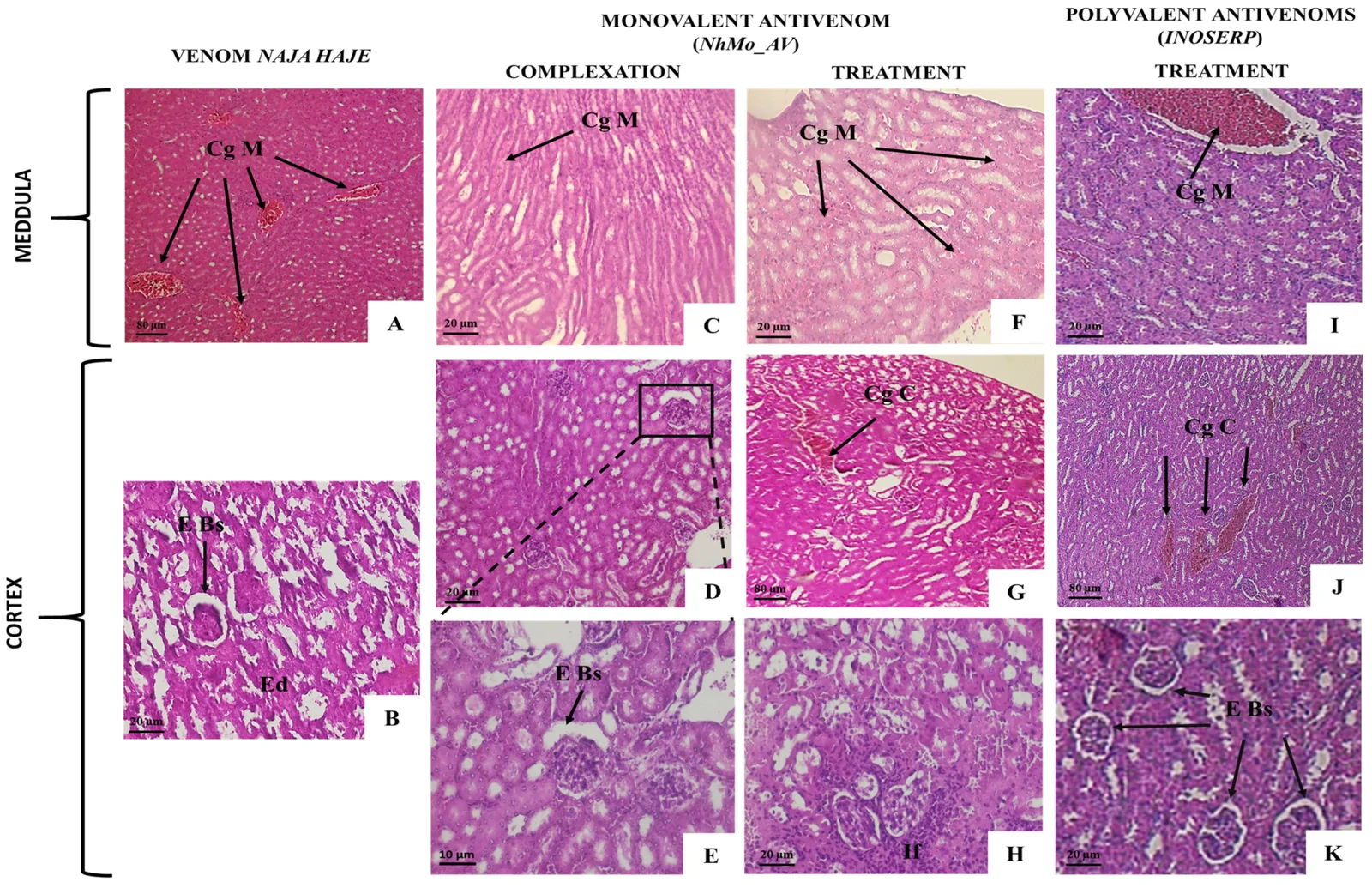
(**A**,**B**)**:** Histopathological examination of renal tissue (cortex and medulla) 6 h after intraperitoneal administration of *Naja haje* venom (½ LD_50_) in mice. (**C**–**E**): Protective effect of monovalent Moroccan antivenom (*NhMo_AV*) on venom-induced morphological changes by complexation method. (**F**–**H**): Envenomated mice treated with monovalent Moroccan antivenom (*NhMo_AV*) after 2 h of *Naja haje* venom injection. (**I**–**K**): Envenomated mice treated with commercialized polyvalent antivenom after 2 h of *Naja haje* venom injection. **CgM**: Medullary congestion, **Ed**: Edema, **CgC**: Congestion of the cortical blood vessels, **EBs**: Enlargement of Bowman’s space, **If**: inflammatory infiltrate.

Group I: The kidneys showed complete resorption of cortical and medullary edema observed in mice injected with *Naja haje* venom. The congestion and dilation of distal and proximal tubules in the cortical region disappeared but remained present in the medullary region (collecting tubules). However, there was slight dilation of Bowman’s spaces (Figure 13C–E and Table 2).

Group II: Mice treated with monovalent antivenom (*NhMo_AV*) two hours after injection of *Naja haje* venom exhibited a protective effect of this antivenom on the kidneys. The cortex architecture was preserved, and the edema was resorbed. However, renal tubules (proximal, distal, and collecting) showed dilation and a polymorphic inflammatory infiltrate (Figure 13F–H and Table 2).

Group III: Histological sections of kidneys treated with commercial polyvalent antivenoms (*Inoserp-MENA*) two hours after injection of *Naja haje* venom showed persistent dilation of Bowman’s spaces and congestion of renal tubules. Partial disappearance of the edema was noted (Figure 13I–K and Table 2).

**At the spleen level**

**Figure 14:**
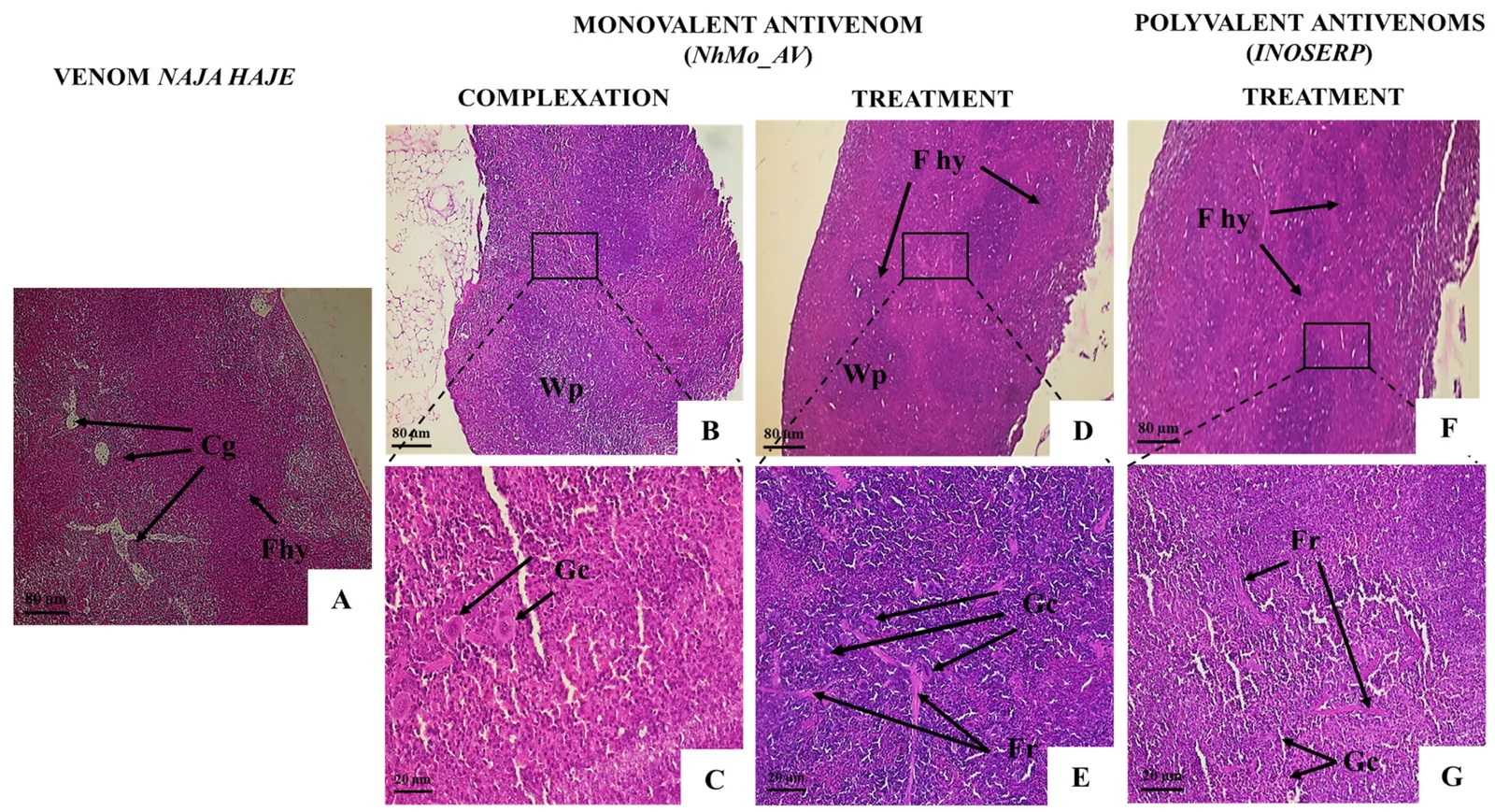
(**A**): Histopathological examination of spleen tissue 6 h after intraperitoneal administration of *Naja haje* venom (½ LD_50_) in mice. (**B**,**C**): Protective effect of monovalent Moroccan antivenom (*NhMo_AV*) on venom-induced morphological changes by complexation method. (**D**,**E**): Envenomated mice treated with monovalent Moroccan antivenom (*NhMo_AV*) after 2 h of *Naja haje* venom injection. (**F**,**G**): Envenomated mice treated with commercialized polyvalent antivenom after 2 h *Naja haje* venom injection. **Wp**: White pulp, **Ar**: Arteries, **Cg**: Congestion, **Gc:** Giant cells, **Fhy:** Follicular hyperplasia.

Group I: The spleen showed complete disappearance of blood vessel congestion, however follicular hyperplasia and giant cells were slightly persistent (Figure 14B,C and Table 2). Group II and III: The spleen tissues showed fibrous remodeling and follicular hyperplasia with an inflammatory reaction rich in giant cells. Vascular congestion was noted to have disappeared (Figure 14D–G and Table 2).

## 4. Discussion

Cobra envenomation is a significant public health issue in many tropical and developing countries, leading to high rates of morbidity and mortality. In North Africa, the cobra *Naja haje* is the most dangerous snake, particularly in Morocco [10]. Its venom is problematic due to its geographic distribution and variable toxicity causing life-threatening symptoms, such as systemic neurotoxicity and local tissue necrosis [17,18,19]. It is well established that the efficacy of antivenom treatments can vary significantly depending on the variability of venoms [7]. To improve current treatments and reduce morbidity and mortality associated with *Naja haje* envenomation in Morocco, our study aims to investigate the physiopathological alterations caused by the venom and assess the neutralization capacity of a developed monovalent Moroccan *Naja haje* antivenom (*NhMo_AV*) and the commercialized polyvalent antivenom *Inoserp-MENA* used in the treatment of snakebite victims in Morocco.

The LD_50_ values of Moroccan *Naja haje* venom are similar between intraperitoneal (0.93 μg/g (95% CI 0.65–1.074 µg/g) (IP) and intravenous (0.80 μg/g (95% CI 0.76–1.035 µg/g) (IV) routes, indicating that the molecules responsible for its lethality are low molecular weight toxins that can quickly diffuse into the bloodstream, with a bioavailability of approximately 100%. Compared to other neurotoxic cobras found in sub-Saharan Africa, such as *Naja haje* from Nigeria, *Naja pallida* from Tanzania, and the forest cobra *Naja melanoleuca* from Uganda. The LD_50_ results for these cobras, determined by intravenous injection, range from 0.6 µg/g (95% CI 0.535–0.649 µg/g) to 0.66 µg/g (95% CI 0.49–0.92 µg/g). As variants of the same species are found in different geographic regions, our venom’s LD_50_ results suggest it is less toxic than its African counterparts [30,31].

The SDS-PAGE analysis of *Naja haje* venom indicated that the venom contains mostly molecules with low molecular weights, below 14 KD and the densitometer analysis revealed that these proteins make up around 80% of the total venom. These results correlate with the toxicity findings, which indicate that the primary toxins responsible for mortality in cobra *Naja haje* envenomation are also low molecular weight toxins [32]. These proteins are generally represented by the three-finger toxins (3FTx) as the major components of the gross venom 58% of which are mainly cytotoxins (54% of all 3FTx detected in *Naja haje* venom), neurotoxins (short neurotoxins, weak neurotoxins, and muscarinic toxins), and cardiotoxins [10,33]. In addition, there are also other components belonging to the non-enzymatic protein families, such as cysteine-rich secretory proteins (CRISPs) (10% of total venom proteins), venom nerve growth factors (vNGF) (5% of total venom proteins), and enzymatic families such as snake venom metalloproteinase (SVMP), acetylcholinesterase (AChE), amino acid oxidase (LAAO), and phospholipase A_2_ (PLA_2_). The overall pathophysiological effect of *Naja haje* venom envenomation results from the combined action of several toxins acting synergistically or additively on different vital organs, complicating the development of specific and effective treatments [19]. The rapid onset of symptoms just minutes after venom injection can be attributed to the low molecular weight of the toxic molecules responsible for the venom’s toxicity. These symptoms primarily included agitation (jumping) and respiratory distress (characterized by tachypnea and dyspnea), which have previously been observed in a study conducted by Demirel and colleagues [34]. These symptoms show that *Naja haje* venom has a potent neurotoxic effect on mice.

Macroscopically, the injection of a sublethal dose of *Naja haje* venom in mice type Swiss (*n* = 4, 20 ± 2 g) showed brain damage as edema accompanied by congestion of blood vessels and infiltrate made of inflammatory cells mainly leucocytes. This can be interpreted by the composition of the venom *Naja haje,* which is rich in non-enzymatic proteins such as neurotoxins, inducing a neurotoxic effect by attacking the central and peripheral nervous system. Besides neurotoxicity, *Naja haje* was found to cause cardiovascular disturbance, myotoxicity, cytotoxicity, and nephrotoxicity in animals and in vitro studies [10,35].

In the lung tissue, the venom of Moroccan *Naja haje* caused alveolar structure disorganization, characterized by a thickening of the alveolar walls. The edema interstitial and congestion with blood vessel dilation have also been observed. Similarly, a study on Sri Lankan *Naja naja* venoms in mice showed vascular abnormalities such as capillary congestion, irregular capillary endothelium, arteritis, and a thickening of the walls and alveolar spaces alveolar, thus an infiltration of inflammatory cells into the interstitial tissue [36]. A. Al-Mamun also reported severe changes in the albino mice (26–30 g) injected with ½ LD_50_ *Naja naja* venom at the pulmonary level such as significant inflammatory cell infiltration and interstitial edema [37]. The observation of the accumulation of leukocytes (neutrophils and macrophages) indicates the neurotoxic effect of Phospholipase A_2_ on the lung tissue [36]. Edematous swelling and pulmonary infiltrates can block alveolar airspaces, disrupting gas exchange and pulmonary mechanics, ultimately leading to respiratory failure. These findings highlight the harmful effects of cobra and *Naja naja* venom on lung tissue, potentially leading to serious respiratory complications.

Compared to the cardiac muscles of control mice, injection of Moroccan *Naja haje* venom results in the alteration of cardiac cells. The myocardial fibers lose their striations, and signs of edema, such as increased inter-fiber space and vascular congestion, are evident. These alterations have also been observed in other studies on envenomation by cobras, including *Naja nigricollis*, *Naja naja,* and *Naja sputatrix* and reported that [36,38,39]. These tissue alterations may be attributed to cardiotoxins, which are responsible for severe tissue necrosis and systolic cardiac arrest in envenomed victims. For example, the venom cardiotoxin *Naja naja*—a low molecular weight polypeptide- causes severe inflammation and congestion leading to the destruction of the membrane structure of heart myocytes, and cardiotoxin (CTX) of the venom *Naja sputatrix* producing myonecrosis [40].

Liver damage is one of the most common and serious symptoms of the envenomation of the cobra snake [41]. Microscopic analysis of the livers of mice envenomed mice revealed alterations in liver tissue characterized by ballooning and cytoplasmic vacuolation of hepatocytes and dilation of the sinusoidal vessel. Studies have reported envenomation with *Naja naja* [36], the royal coba *Ophiophagus hannah* [42], and *Walterinnesia aegyptia* [43]. According to the results of Tohamy and coworkers’ study [44], the effect of PLA_2_ from the venom *Naja haje* could be among the factors responsible for the rupture of liver cell membranes, as seen in the present study. The other studies reported that the venom metalloproteinase *Walterinnesia aegyptia* binds directly to the GPIIb/GPIIIa receptor present on the surface of platelets, resulting in hemorrhage [43]. Histological changes in the liver can be assessed in vitro by evaluating the activity of liver enzymes alanine transaminase (ALT) and aspartate transaminase (AST). Studies have shown a significant increase in these enzymes in animals inoculated by the venoms *Naja naja* and *Walterinnesia aegyptia* and *Naja nigricollis*, indicating the destruction of hepatic cell organelles and the intracellular release of these enzymes [43,44].

After being injected with a sublethal dose of *Naja haje* venom, mice showed a disorganized structure of the renal parenchyma. This was characterized by dilation of Bowman’s space, edema in the cortex and medulla, and congestion and dilation of the distal and proximal tubules. Renal alterations are similar to those observed in *Naja nigricollis*, *Naja Haje,* and *Ophiophagus hannah* venoms have been reported, which induce morphological changes in the renal glomeruli resulting in complete lysis of renal corpuscles with rupture of Bowman capsules and acute tubular necrosis [39,42]. These renal abnormalities can be attributed to the higher absorption of the venom in the kidneys of the animals, as previously demonstrated with *Naja nigricollis* venom leading to renal abnormalities. The action of the venom on the kidneys may be direct or indirect through toxins [45]. An Immunohistochemistry study on the renal tissues of mice envenomed with *Naja nigricollis* venom and using CD34, a marker of endothelial cells, showed the direct effect of the venom on the endothelial cells of the glomerular capillaries [46].

Snakebites can cause severe systemic effects that can lead to permanent damage to vital tissues and death. However, rapid administration of antivenom serums could potentially reverse the systemic effects of snakebites, thus neutralizing permanent changes to vital tissues and preventing death [47]. The World Health Organization (WHO) considers the preclinical evaluation of antivenom efficacy to be the gold standard for lethality neutralization, and it is a routine quality control measure undertaken by laboratories. This ensures that antivenom sera are effective in treating snakebites and can help to prevent unnecessary deaths. In this study, the neutralization capabilities of two antivenoms were evaluated. One antivenom was monospecific, developed specifically against the venom of the Moroccan cobra *Naja haje*. The other antivenom was polyspecific, marketed in Morocco, and developed against the venoms of various snake species, including those belonging to the genera *Echis*, *Cerastes*, *Daboia*, *Bitis,* and *Cobra*. The antivenoms were tested for their ability to neutralize mortality by calculating the median effective dose (ED_50_) and potency (P). Additionally, their capacity to neutralize the observed alterations in different organs of envenomed mice was also assessed. At a dose of 5 LD_50_ of *Naja haje* cobra venom, the monovalent antivenom (*NhMo_AV*) showed a toxicity neutralizing capacity four times higher than the antivenom marketed in Morocco (*Inoserp-MENA*) with a neutralizing power of 1.3 mg/mL. In contrast, the multipurpose commercialized antivenom (*Inoserp-MENA*) has an ED_50_ value of 100 µL and a neutralizing power of 0.32 mg/mL. The monovalent antivenom (*NhMo_AV*) exhibited a toxicity-neutralizing capacity that was four times higher than the antivenom commercialized in Morocco (*Inoserp-MENA*) when tested at a dose of 5 LD_50_ of *Naja haje* cobra venom. *NhMo_AV* showed a neutralizing power of 1.3 mg/mL. In contrast, the multipurpose commercialized antivenom (*Inoserp-MENA*) had an ED_50_ value of 100 µL and a neutralizing power of 0.32 mg/mL.

While the advantages of polyvalent antivenoms, such as the ability to treat snake bites without species identification (para-specific neutralization), are widely recognized, there is ongoing debate regarding their efficacy and potential for adverse effects compared to monovalent anti-venoms [48,49,50]. It is worth noting that, despite the wide distribution of *Naja haje* in Africa and the Middle East, there is currently no specific antivenom available for its envenomation.

In a study by Manson in 2022, the neutralizing capacity of two commercial antivenoms, *VINS™* and *Inoserp™*, against *Naja ashei* venom was investigated in Kenya. The study’s results revealed that both *VINS™* and *Inoserp™* antivenoms could neutralize the lethal and toxic effects of *Naja ashei* venom, although their efficiencies varied. Notably, the *Inoserp™* antivenom required a slightly higher dose than *VINS™* antivenom to achieve the same neutralizing effect. The authors attributed this difference in efficacy to the contrasting protein content of the two antivenom batches: *Inoserp™* (28.213 mg/mL) and *VINS™* (98.913 mg/mL) [51]. Resieri and colleagues made observations, highlighting the high efficacy of a monospecific antivenom, which aligned with its previously reported clinical effectiveness. They also speculated that discrepancies in protein concentrations among antivenom batches could account for the observed variations. Other studies on the ability of commercial polyspecific and monospecific antivenoms to neutralize the lethality of several African cobras have been reported and make it clear that when antivenoms developed from the venom of one geographical region are used to treat patients in another, efficacy is significantly reduced, depending largely on the source of the venom used to immunize the horses [52]. As has been demonstrated by the study of the authors of this study [45], showing that Indian *PAV* had low potency in neutralizing the lethality of *Naja kaouthia* venom from West Bengal in eastern India and was completely unsuccessful in neutralizing the lethality of the same venom from Arunachal Pradesh in Northeast India.

The physiological lesions caused by *Naja haje* venom, including vascular congestion in the heart and kidneys, pulmonary and renal edema, cytoplasmic vacuolization of hepatocytes in the liver, and infiltration of inflammatory cells in the brain and spleen, showed signs of improvement in the tissue of the groups treated with the monospecific antivenom (*NhMo_AV*). However, the commercial antivenom, *Inoserp-MENA*, showed the persistence of these cardiac, renal, hepatic, and pulmonary lesions. A study was conducted to evaluate the effectiveness of polyvalent antivenom on the biochemical and histological disorders induced by *Naja haje* cobra venom. The results showed a decrease in biochemical parameters such as ALT, AST, ALP, and LDH, as well as a reduction in lesions in the examined organs (liver, spleen, and heart). These organs appeared more or less normal with very few remaining anomalies [53] something that was not observed in our study.

## 5. Conclusions

This study aimed to histopathologically compare the efficacy of two types of antivenoms: one monospecific, directed solely against the venom of the cobra *Naja haje,* and another one marketed to treat envenomation by endemic species in the Middle East and North Africa, including the cobra *Naja haje*. For this task, we evaluated the toxic potency of this species by calculating its LD_50_ and reproducing the conditions of a real envenomation in mice. We then observed the different induced symptomatologies and performed a histological examination of vital organs (brain, liver, lungs, kidneys, heart, and spleen) to assess the damage caused by *Naja haje* venom before and after treatment with both antivenoms. Our results, as well as previous work, revealed significant differences between the two antivenoms in terms of toxicity neutralizing and severe signs of envenomation, mechanism of action, and individual effect on each vital organ, showing that high doses of polyvalent antivenom were necessary for the treatment of *Naja haje* envenomation in contrast to the monospecific antivenom. We also found a geographical variation of the cobra *Naja haje,* which leads to different histological lesions, including systemic hemorrhages in vital organs in mice injected with venom from Egypt, which are not observed in mice injected with *Naja haje* venom from Morocco. This difference can reduce the effectiveness of the antivenom marketed in the Middle East. Therefore, it is necessary to establish a more effective treatment plan, primarily neutralizing the effects of the main actors of *Elapidae* envenomation in Morocco. We hope these results will improve the management of patients envenomed by the cobra *Naja haje* in the studied regions.

## Figures and Tables

**Table 1:** Determination of the LD_50_ of the venom *Naja haja*, using IV and IP injection routes, with 95% confidence intervals and calculated by non-linear regression.

	*Naja haje* Venom		
Injection Route	µg/Mouse	µg/g	Ratio (IV/IP)
Intravenous IV	18.6 (15.2–20.7)	0.93 (0.76–1.035)	1.16
Intraperitoneal IP	16.05 (13.16–21.49)	0.80 (0.65–1.074)	

**Table 2:** Major histopathological changes in vital organs induced by *Naja haje* venom and neutralized by monospecific *NhMo_AV* and commercialized antivenom. Grade: −: absent; +: mild; ++; moderate; and +++: severe.

Microscopic Observation	Groups				
	Control	*Naja haje*	Monovalent Moroccan *Naja haje* Antivenom (*NhMo_AV*)		Commercialzed Antivenom (*Inoserp-MENA)*
			Complexation	Treatment	Treatment
Brain Tissue					
Cerebral edema	−	++	+	+	++
Inflammatory cell infiltrate	−	++	−	−	+
Vascular congestion	−	+++	+	+	++
Cardiac Tissue					
Loss of striations in the myocardial fibers	−	++	−	−	−
Interstitial edema	−	+++	−	+	++
Myocardial cells Hypertrophy	−	++	−	+	+
Vascular congestion and dilation	−	+++	+	+	++
Lung Tissue					
Thickening of the alveolar walls	−	+++	+	+	++
Pulmonary edema	−	+++	−	+	++
Emphysema	−	+	+	+	++
Polymorphic inflammatory cells	−	++	−	+	+
Vascular congestion and dilation	−	+++	+	++	++
Hepatic Tissue					
Ballooning of hepatocytes	−	+++	+	+	++
Cytoplasmic vacuolation of hepatocyte	−	++	−	+	++
Dilation of the sinusoidal vessels	−	++	+	+	+
Congestion and dilation of central venous	−	+++	+	+	++
Polynuclear inflammatory cells	−	++	−	+	++
Kidney Tissue					
Dilation of Bowman’s space	−	++	+	+	++
Cortical and medullary edema	−	+++	−	−	+
Congestion and dilatation of distal and proximal tubules	−	+++	−	++	++
Cell hypertrophy	−	++	+	+	+
Infiltration of polymorphic inflammatory cells	−	+	+	+	++
Splenic Tissue					
Follicular hyperplasia	−	++	+	+	+
Presence of giant cells	−	++	+	++	++
Congestion	−	++	−	−	−
Infiltration of inflammatory cells	−	+	−	+	+

**Table 3:** Neutralizing potencies of the monospecific antivenom anti-*Naja haje* and commercial antivenom were expressed in µL/mouse and the protective activity (P) in mg/mL.

*Naja haje* Venom			Antivenom Neutralization					
i.p LD_50_^a^ (μg/g)		Challenge Dose	Antivenom	ED_50_^b^ (μL)	ER_50_^c^ (mg/mL)	P ^d^ (mg/mL)	Antivenom Concentration (mg/mL)	Normalized Potency ^e^ (mg/g)
*Naja haje*	0.80	5 LD_50_	*NhMo_AV*	67.2 (50.45–70.8)	1.19 (1.60–1.13)	0.95	74.5 ± 4.70	12.84
		3 LD_50_	*Inoserp-MENA*	100 (112.5–122.5)	0.48 (0.428–0.39)	0.32	20 ± 3.45	16.05

^a^ Median lethal dose, defined as the dose of venom (μg/g) at which 50% of mice were dead. **^b^** Median effective dose, defined as the dose of antivenom (μL) at which 50% of mice survived. **^c^** Median effective ratio, defined as the ratio of venom (mg) to the volume of antivenom (mL) at which 50% of mice survived. **^d^** Potency, defined as the amount of venom (mg) completely neutralized by one ml of antivenom (mL). **^e^** Normalized P, defined as the amount of venom (mg) completely neutralized per unit amount of antivenom protein (g).

## Data Availability

Not applicable.

## References

[1] Gutiérrez J.M., Calvete J.J., Habib A.G., Harrison R.A., Williams D.J., Warrell D.A. (2017). Snakebite envenoming. Nat. Rev. Dis..

[2] Chippaux J.P. (2017). Snakebite envenomation turns again into a neglected tropical disease!. J. Venom. Anim. Toxins Incl. Trop. Dis..

[3] Chippaux J.P. (2011). Estimate of the burden of snakebites in sub-Saharan Africa: A meta-analytic approach. Toxicon.

[4] Benjamin J.M., Abo B.N., Brandehoff N. (2020). Review Article: Snake Envenomation in Africa. Curr. Trop. Med. Rep..

[5] Brito J.C., Fahd S., Geniez P., Martínez-Freiría F., Pleguezuelos J.M., Trape J.F. (2011). Biogeography and conservation of viperids from North-West Africa: An application of ecological niche-based models and GIS. J. Arid. Environ..

[6] Wilkins D., Burns D.S., Wilson D., Warrell D.A., Lamb L.E.M. (2018). Snakebites in Africa and Europe: A military perspective and update for contemporary operations. J. R. Army Med. Corps.

[7] Modahl C.M., Roointan A., Rogers J., Currier K., Mackessy S.P. (2020). Interspecific and intraspecific venom enzymatic variation among cobras (*Naja* sp. and *Ophiophagus hannah*). Comp. Biochem. Physiol. Part C Toxicol. Pharmacol..

[8] Borja M., Lazcano D., Martínez-Romero G., Morlett J., Sánchez E., Cepeda-Nieto A.C., Garza-García Y., Zugasti-Cruz A. (2013). Intra-specific Variation in the Protein Composition and Proteolytic Activity of Venom of Crotalus lepidus morulus from the Northeast of Mexico. Copeia.

[9] Chippaux J.P., White J., Habib A.G., Brent J., Burkhart K., Dargan P., Hatten B., Megarbane B., Palmer R. (2017). African Snakes. Critical Care Toxicology.

[10] Malih I., Ahmad Rusmili M.R., Tee T.Y., Saile R., Ghalim N., Othman I. (2014). Proteomic analysis of Moroccan cobra Naja haje legionis venom using tandem mass spectrometry. J. Proteom..

[11] Karam H., Shaaban E., Fahmy A., Zaki H., Kenawy S. (2021). Improvement of *Naja haje* snake antivenom production using gamma radiation and a biotechnological technique. Toxin Rev..

[12] Berling I., Isbister G.K. (2015). Hematologic Effects and Complications of Snake Envenoming. Transfus. Med. Rev..

[13] Lomonte B., Križaj I. (2021). Snake Venom Phospholipase A2 Toxins. Handbook of Venoms and Toxins of Reptiles.

[14] Ajisebiola B.S., Adeniji O.B., James A.S., Ajayi B.O., Adeyi A.O. (2022). Naja nigricollis venom altered reproductive and neurological functions via modulation of pro-inflammatory cytokines and oxidative damage in male rats. Metab. Open.

[15] Brunda G., Sashidhar R.B., Sarin R.K. (2006). Use of egg yolk antibody (IgY) as an immunoanalytical tool in the detection of Indian cobra (*Naja naja naja*) venom in biological samples of forensic origin. Toxicon.

[16] Tasoulis T., Isbister G. (2017). A Review and Database of Snake Venom Proteomes. Toxins.

[17] Wong K.Y., Tan C.H., Tan K.Y., Quraishi N.H., Tan N.H. (2018). Elucidating the biogeographical variation of the venom of *Naja naja* (*spectacled cobra*) from Pakistan through a venom-decomplexing proteomic study. J. Proteom..

[18] Tan N.H., Wong K.Y., Tan C.H. (2017). Venomics of Naja sputatrix, the Javan spitting cobra: A short neurotoxin-driven venom needing improved antivenom neutralization. J. Proteom..

[19] Palasuberniam P., Chan Y.W., Tan K.Y., Tan C.H. (2021). Snake Venom Proteomics of Samar Cobra (*Naja samarensis*) from the Southern Philippines: Short Alpha-Neurotoxins as the Dominant Lethal Component Weakly Cross-Neutralized by the Philippine Cobra Antivenom. Front. Pharmacol..

[20] Hansson E., Sasa M., Mattisson K., Robles A., Gutiérrez J.M. (2013). Using Geographical Information Systems to Identify Populations in Need of Improved Accessibility to Antivenom Treatment for Snakebite Envenoming in Costa Rica. PLoS Negl. Trop. Dis..

[21] WHO Issues New Recommendation on Antivenom for Snakebites. https://www.who.int/news/item/19-08-2018-who-issues-new-recommendation-on-antivenom-for-snakebites.

[22] Becker E.L. (1953). Antigen-antibody reactions in gels. Fed. Proc..

[23] Chavez-Olortegui C., Lopes C.S., Cordeiro F.D., Granier C., Diniz C.R. (1993). An enzyme linked immunosorbent assay (ELISA) that discriminates between Bothrops atrox and Lachesis muta muta venoms. Toxicon.

[24] Khoobdel M., Fasaei B.N., Salehi T.Z., Khosravi M., Taheri M., Koochakzadeh A., Masihipour B., Motedayen M., Akbari S. (2013). The production of monovalent and anti-idiotype antivenom against Mesobuthus eupeus (*Scorpionida: Buthidae*) venom in rabbits. Toxicon.

[25] Laemmli U.K. (1970). Cleavage of Structural Proteins during the Assembly of the Head of Bacteriophage T4. Nature.

[26] Oukkache N., El Jaoudi R., Ghalim N., Chgoury F., Bouhaouala B., El Mdaghri N., Sabatier J.-M. (2014). Evaluation of the Lethal Potency of Scorpion and Snake Venoms and Comparison between Intraperitoneal and Intravenous Injection Routes. Toxins.

[27] Vollmer R.T. (1983). Theory and Practice of Histological Techniques. JAMA.

[28] Ross M.H., Pawlina W. (2006). Histology.

[29] Trevors J.T. (1986). A BASIC Program for Estimating LDso Values Using the IBM-PC. Bull. Environ. Contam. Toxicol..

[30] Tan K.Y., Tan C.H., Sim S.M., Fung S.Y., Tan N.H. (2016). Geographical venom variations of the Southeast Asian monocled cobra (*Naja kaouthia*): Venom-induced neuromuscular depression and antivenom neutralization. Comp. Biochem. Physiol. Part C Toxicol. Pharmacol..

[31] World Health Organization (2017). WHO Expert Committee on Biological Standardization (2016: Geneva S. WHO Expert Committee on Biological Standardization, Sixty-Seventh Report.

[32] Mukherjee A.K., Maity C.R. (2002). Biochemical composition, lethality and pathophysiology of venom from two cobras—*Naja naja* and *N. kaouthia*. Comp. Biochem. Physiol. Part B Biochem. Mol. Biol..

[33] Lafnoune A., Lee S.-Y., Heo J.-Y., Gourja I., Darkaoui B., Abdelkafi-Koubaa Z., Chgoury F., Daoudi K., Chakir S., Cadi R. (2021). Anti-Cancer Effect of Moroccan Cobra Naja haje Venom and Its Fractions against Hepatocellular Carcinoma in 3D Cell Culture. Toxins.

[34] Demirel M.A., Alcigir M.E., Ozkan O., Turkmen M.B. (2021). The effects of antivenom administrations on the brain tissue of experimentally envenomed pregnant rats and their pups with *Androctonus crassicauda* scorpion venom during organogenesis period. Toxicon.

[35] Naoual O., Rachid E., Sebastien L., Salma C., Chafi Q.F., Abdelaziz H., Georges M. (2019). Snake bites in morocco: Progress and challenges. Adv. Toxicol. Toxic. Eff..

[36] Dissanayake D.S.B., Thewarage L.D., Waduge R.N., Ranasinghe J.G.S., Kularatne S.A.M., Rajapakse R.P.V.J. (2018). The Venom of Spectacled Cobra (Elapidae: *Naja naja*): In Vitro Study from Distinct Geographical Origins in Sri Lanka. J. Toxicol..

[37] Al-Mamun M.A., Hakim M.A., Zaman M.K., Hoque K.M.F., Ferdousi Z., Reza M.A. (2015). Histopathological Alterations Induced by *Naja naja* Crude Venom on Renal, Pulmonary and Intestinal Tissues of Mice Model. Biotechnol. J. Int..

[38] Cher C.D.N., Armugam A., Zhu Y.Z., Jeyaseelan K. (2005). Molecular basis of cardiotoxicity upon cobra envenomation. Cell. Mol. Life Sci..

[39] Wang C.-H., Liu J.-H., Lee S.-C., Hsiao C.-D., Wu W.-G. (2006). Glycosphingolipid-facilitated Membrane Insertion and Internalization of Cobra Cardiotoxin. J. Biol. Chem..

[40] Hung D.Z., Liau M.Y., Lin-Shiau S.Y. (2003). The clinical significance of venom detection in patients of cobra snakebite. Toxicon.

[41] Adzu B., Abubakar M.S., Izebe K.S., Akumka D.D., Gamaniel K.S. (2005). Effect of Annona senegalensis rootbark extracts on Naja nigricotlis nigricotlis venom in rats. J. Ethnopharmacol..

[42] Al-Ani I., Ismail S., Maung K., Oothuman P., Al-Mahmood S. (2017). Histological study on the protective effects of Tamarind seed extract on Cobra venom in mice. Asian J. Pharm. Clin. Res..

[43] Al-Sadoon M.K., Orabi G.M., Badr G. (2013). Toxic Effects of Crude Venom of a Desert Cobra, Walterinnesia aegyptia, on Liver, Abdominal Muscles and Brain of Male Albino Rats. Pak. J. Zool..

[44] Tohamy A.A., Mohamed A.F., Abdel Moneim A.E., Diab M.S.M. (2014). Biological effects of Naja haje crude venom on the hepatic and renal tissues of mice. J. King Saud Univ. Sci..

[45] Ding Z.-H., Xu L.-M., Wang S.-Z., Kou J.-Q., Xu Y.-L., Chen C.-X., Yu H.-P., Qin Z.-H., Xie Y. (2014). Ameliorating Adriamycin-Induced Chronic Kidney Disease in Rats by Orally Administrated Cardiotoxin from *Naja naja atra* Venom. Evid. Based Complement. Altern. Med..

[46] Abdel-Ghani L., El-Asmer M., Abuzinadah O., Abbas A., Rahmy T. (2010). Histological and immunohistochemical studies on the nephrotoxic effects of naja nigricollis snake venom. Egypt. J. Nat. Toxins.

[47] Rivel M., Solano D., Herrera M., Vargas M., Villalta M., Segura Á., Arias A.S., León G., Gutiérrez J.M. (2016). Pathogenesis of dermonecrosis induced by venom of the spitting cobra, Naja nigricollis: An experimental study in mice. Toxicon.

[48] Senji Laxme R.R., Khochare S., De Souza H.F., Ahuja B., Suranse V., Martin G., Whitaker R., Sunagar K. (2019). Beyond the ‘big four’: Venom profiling of the medically important yet neglected Indian snakes reveals disturbing antivenom deficiencies. PLoS Negl. Trop. Dis..

[49] Sandesha V.D., Darshan B., Tejas C., Girish K.S., Kempaiah K. (2022). A comparative cross-reactivity and paraspecific neutralization study on Hypnale hypnale, Echis carinatus, and Daboia russelii monovalent and therapeutic polyvalent anti-venoms. PLoS Negl. Trop. Dis..

[50] Tan K.Y., Wong K.Y., Tan N.H., Tan C.H. (2020). Quantitative proteomics of Naja annulifera (sub-Saharan snouted cobra) venom and neutralization activities of two antivenoms in Africa. Int. J. Biol. Macromol..

[51] Manson E.Z., Kyama M.C., Gikunju J.K., Kimani J., Kimotho J.H. (2022). Evaluation of lethality and cytotoxic effects induced by Naja ashei (large brown spitting cobra) venom and the envenomation-neutralizing efficacy of selected commercial antivenoms in Kenya. Toxicon X.

[52] Shashidharamurthy R., Kemparaju K. (2007). Region-specific neutralization of Indian cobra (*Naja naja*) venom by polyclonal antibody raised against the eastern regional venom: A comparative study of the venoms from three different geographical distributions. Int. Immunopharmacol..

[53] Shaban E.A., Hafez M.N. (2003). Ability of Gamma-Irradiated Polyvalent Antivenin to Neutralize the Toxicity of the Egyptian Cobra (Naja haje) Venom. Egypt J. Hosp. Med..

