# Correction: Khourcha et al. Assessing the Efficacy of Monovalent and Commercialized Antivenoms for Neutralizing Moroccan Cobra *Naja haje* Venom: A Comparative Study. *Trop. Med. Infect. Dis.* 2023, *8*, 304

**DOI:** 10.3390/tropicalmed11080233

**Published:** 2026-08-19

**Authors:** Soukaina Khourcha, Ines Hilal, Iatimad Elbejjaj, Mehdi Karkouri, Amal Safi, Abdelaziz Hmyene, Naoual Oukkache

**Affiliations:** 1 Laboratory of Venoms and Toxins, Pasteur Institute of Morocco, Casablanca 20360, Morocco; drineshilal@gmail.com (I.H.); naoual.oukkache@pasteur.ma (N.O.); 2 Laboratory of Biochemistry, Environment and Food Technology, Faculty of Sciences and Technologies of Mohammedia, Hassan II University, Mohammedia 20650, Morocco; amalsafi88@gmail.com (A.S.); hmyeneaziz2002@yahoo.fr (A.H.); 3 Laboratory of Pathological Anatomy, University Hospital Center Ibn Rochd, Casablanca 20250, Morocco; madowine.elbejaj@gmail.com (I.E.); m.karkouri@chucasa.ma (M.K.)

There was an error in the original publication [1]. The cage dimensions, floor area per animal, humane endpoints, and euthanasia method were not explicitly reported in the original Materials and Methods section.

A correction has been made to the Materials and Methods Section, 2.7. Animals and Ethics Statement:

1. Standard cages (floor area):

Mice were housed in standard cages measuring 42.5 cm × 26.6 cm × 18.5 cm, corresponding to a total floor area of approximately 1130 cm^2^, with four animals per cage (approximately 282 cm^2^ per animal).

2. Humane endpoint and euthanasia method:

Animals were closely monitored throughout the experiments, and predefined humane endpoints were applied, including severe distress or moribund state.

Mice were euthanized by CO_2_ inhalation using a gradual-fill method, in accordance with current ethical recommendations. Death was confirmed by the absence of respiratory movements and lack of response to external stimuli.

The authors state that the scientific conclusions are unaffected. This correction was approved by the Academic Editor. The original publication has also been updated.
